# Molecular Apomorphies in the Secondary and Tertiary Structures of Length-Variable Regions (LVRs) of 18S rRNA Shed Light on the Systematic Position of the Family Thaumastellidae (Hemiptera: Heteroptera: Pentatomoidea)

**DOI:** 10.3390/ijms24097758

**Published:** 2023-04-24

**Authors:** Jerzy A. Lis

**Affiliations:** Institute of Biology, University of Opole, Oleska 22, 45-052 Opole, Poland; cydnus@uni.opole.pl

**Keywords:** *Thaumastella*, cydnoid complex, phylogeny, molecular apomorphies

## Abstract

The *SSU nrDNA*, a small subunit of the nuclear ribosomal DNA (coding *18S rRNA*), is one of the most frequently sequenced genes in molecular studies in Hexapoda. In insects, including true bugs (Hemiptera: Heteroptera), only its primary structures (i.e., aligned sequences) are predominantly used in phylogenetic reconstructions. It is known that including RNA secondary structures in the alignment procedure is essential for improving accuracy and robustness in phylogenetic tree reconstruction. Moreover, local plasticity in rRNAs might impact their tertiary structures and corresponding functions. To determine the systematic position of Thaumastellidae within the superfamily Pentatomoidea, the secondary and—for the first time among all Hexapoda—tertiary structures of 18S rRNAs in twelve pentatomoid families were compared and analysed. Results indicate that the shapes of the secondary and tertiary structures of the length-variable regions (LVRs) in the 18S rRNA are phylogenetically highly informative. Based on these results, it is suggested that the Thaumastellidae is maintained as an independent family within the superfamily Pentatomoidea, rather than as a part of the family Cydnidae. Moreover, the analyses indicate a close relationship between Sehirinae and Parastrachiidae, expressed in morpho-molecular synapomorphies in the predicted secondary and tertiary structures of the length-variable region L (LVR L).

## 1. Introduction

In recent decades, studies on ribosomal RNA molecules—especially the nuclear *18S rRNA* sequences—have significantly improved the quality of phylogenetic reconstructions in insects [1,2,3,4,5,6,7]. Analyses based on the sequence alignment using relevant models for nucleotide substitution are now widely accepted in molecular systematics [7,8,9,10,11,12,13]. However, the increase in the application of rRNA secondary structures in hexapod phylogenetics is less pronounced and, as far as the tertiary structures are concerned, progress is still not satisfactory [4,5,14,15,16,17,18,19].

Unfortunately, the usefulness of the secondary structures of nuclear *18S rRNA* sequences in resolving the phylogenetic problems in Hemiptera in general, and Heteroptera in particular, has only been considered sporadically [9,10,19,20,21]. Nevertheless, the results have been promising, suggesting that the secondary structures of *18S rRNA* could be a crucial new parameter in insect phylogenetic reconstructions. The heteropteran superfamily Pentatomoidea is the only taxon in which such analyses have been conducted comprehensively [19,20]. Unfortunately, only one study [20] considered the pentatomoid family Thaumastellidae, the within-subfamily systematic position of which is ambiguous.

In contrast to the secondary structures, tertiary rRNA structures have never been used in resolving the problems of relationships among taxa in Hemiptera in particular, or in Hexapoda in general.

**Figure A1:**
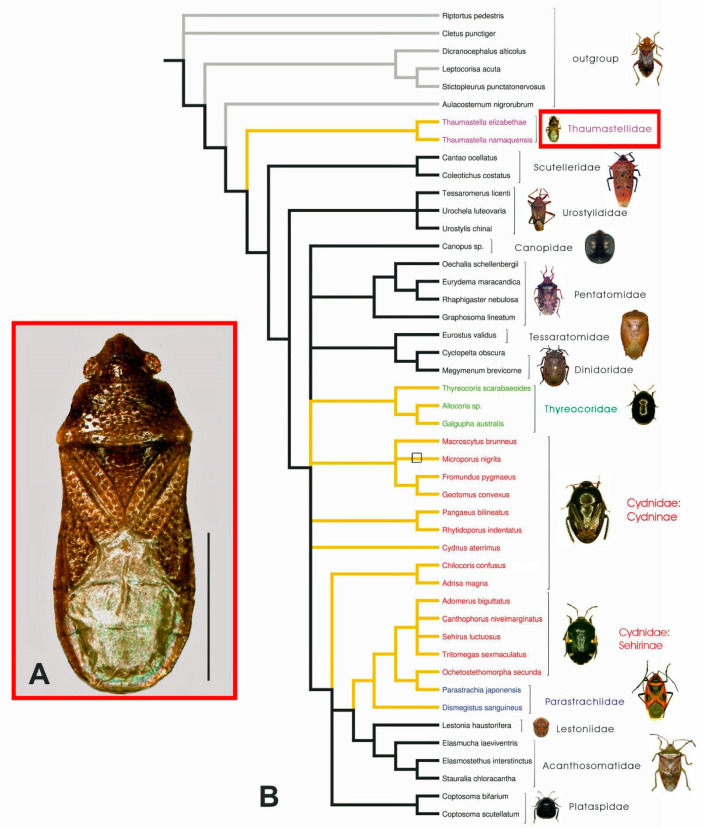
(**A**) *Thaumastella aradoides*, general habitus (oryg.). (**B**) Phylogenetic tree of the superfamily Pentatomoidea selected as the base for the analysis (after [7], modified). Scale bar = 1 mm (**A**).

The Thaumastellidae is a small pentatomoid family containing only one described genus—*Thaumastella* Horváth, 1896—and three species [22,23,24,25,26,27]. The individuals of *T*. *aradoides* Horváth, 1896 (North and East Africa, Near East), bearing macro- or sub-macropterous hemelytra (Figure A1A), can fly. In contrast, individuals of two Afrotropical species (Republic of South Africa, Namibia), i.e., *T*. *namaquensis* Schaefer and Wilcox, 1971 and *T*. *elizabethae* Jacobs, 1989 are completely flightless, exhibiting a staphylinoid type of forewing [22,24,25,26]. Although the species of Thaumastellidae have a limited distribution area and their specimens have been sporadically collected [22,24,25,28,29], the family is significant from a phylogenetic point of view.

*Thaumastella* was initially assigned to the subfamily Artheneinae of the family Lygaeidae [30]. Due to many morphological characters incompatible with those that are diagnostic for the Artheneinae, it was then assigned to a subfamily of its own, i.e., Thumastellinae, within Lygaeidae [22]. Later on, Štys [23], based on a detailed morphological study of *T*. *aradoides*, upgraded Thumastellinae to the family level and transferred it from the Lygaeidae (Lygaeoidea) to the Pentatomoidea. He also suggested the Thaumastellidae to be an early offshoot of the main pentatomoid stock and the sister taxon to all remaining Pentatomoidea [23]. Since then, the systematic position of *Thaumastella* as a part of the superfamily Pentatomoidea has never been challenged, and the genus has usually been considered very primitive (sometimes even the most primitive) within the entire superfamily [7,23,24,25,31,32,33,34].

Most importantly, the interpretation of its systematic position has varied significantly depending on the author and the type of analysis. Three competing hypotheses thus exist: *Thaumastella* as (1) a taxon of the family rank, being sister to all other Pentatomoidea [7,20,32,33,34,35,36]; (2) closely related to the Cydnidae [24,31,37,38,39,40,41,42,43,44,45]; or (3) a subfamily in the broadly conceived Cydnidae (‘cydnoid complex’ sensu Lis et al., 2017; Cydnidae sensu Schuh and Weirauch, 2020) [25,27,46,47,48,49].

In recent molecular studies, where the systematic position of Thaumastellidae was considered [7,20,35,36,40], the genes coding ribosomal RNA—i.e., mitochondrial *16S rDNA* and nuclear *18S rDNA* and *28S rDNA*—were predominantly utilised. I decided to focus the molecular analyses on the *18S rDNA* gene (also known as SSU—a small subunit of the nuclear ribosomal DNA) because it was always the only gene with a complete sequence used in this type of study. Moreover, the yield of informative structural changes for insect phylogeny is high in *18S rDNA* but poor in *28S* and *16S rDNA* [2,3,50].

The *SSU nrDNA* (coding *18S rRNA*) is one of the most frequently sequenced genes in insect molecular studies [1,2,3,6,7,8,9,10,11,15,19,20,21,35,36,40]. However, in Heteroptera, its primary structure (i.e., aligned sequences) is usually used in phylogenetic reconstructions [6,7,8,9,10,11,13,35,36,40,44,51,52,53]. Nevertheless, as was observed many times [12,15,17,18,19,20,21], the hypervariation of length at multiple positions of this gene can seriously affect the accuracy of alignment of the sequences’ primary structures. Therefore, it is essential to refer to the secondary structures of the sequences during the alignment process, with particular attention to the gene length-variable regions (LVRs) [2,3,7,9,12,15,18,19,20,21]. It has been proven that including RNA secondary structures in the alignment procedure can significantly improve accuracy and robustness in phylogenetic tree reconstruction [7,9,12,17,18,19,20].

It is essential to note that some length-variable regions (LVRs) can serve as autapomorphies or synapomorphies for monophyletic groups recovered during the phylogenetic analysis [3,9,15,19,20,21]. To my knowledge, only in three papers related to the suborder Heteroptera [19,20,21] did the authors extract information from certain length-variable regions (LVRs) of the *18S rRNA* secondary structure and use them to define the possible molecular autapomorphies or synapomorphies in the recovered monophyletic groups of the species. Moreover, it has been suggested [3,54] that the local plasticity in rRNAs (the LVRs’ presence or absence) might impact their tertiary structures and corresponding functions. Unfortunately, the *18S nuclear rRNA* tertiary structures have been considered only sporadically in phylogenetic analyses in Hexapoda [2,3] and never in Hemiptera.

As was shown for other eukaryotic rRNAs [5,12,16,55], the tertiary structure exhibits evolutionary properties detectable neither within the primary (i.e., sequence) nor in the secondary structure. Therefore, the results of analyses considering rRNA tertiary structures for phylogenetic considerations seem very promising [2,3,5,55]. Thus, I regard it as crucial to consider the ribosomal RNA secondary and tertiary structures during phylogenetic analyses based on molecular data.

The primary purpose of the present study aims to shed light on the systematic position of the family Thaumastellidae using molecular synapomorphies in the secondary and—for the first time—the tertiary structures of length-variable regions (LVRs) of *18S rRNA*.

## 2. Results

### 2.1. 18S rRNA Secondary Structure Models

The secondary structure models of the *18S rRNA* gene were predicted for 46 analysed taxa, including fifteen consensus species (Table S1, Files S1 and S2). The prediction included six species of the superfamily Coreoidea (representing the outgroup) and forty species of the superfamily Pentatomoidea constituting the ingroup. The latter covered fourteen species of the family Cydnidae; four species of the Pentatomidae; three species for each of the Acanthosomatidae, Thyreocoridae and Urostylididae; two species for each of the Dinidoridae, Plataspidae, Parastrachiidae, Scutelleridae and Thaumastellidae; and a single species for the Canopidae, Lestoniidae and Tessaratomidae.

**Figure 1:**
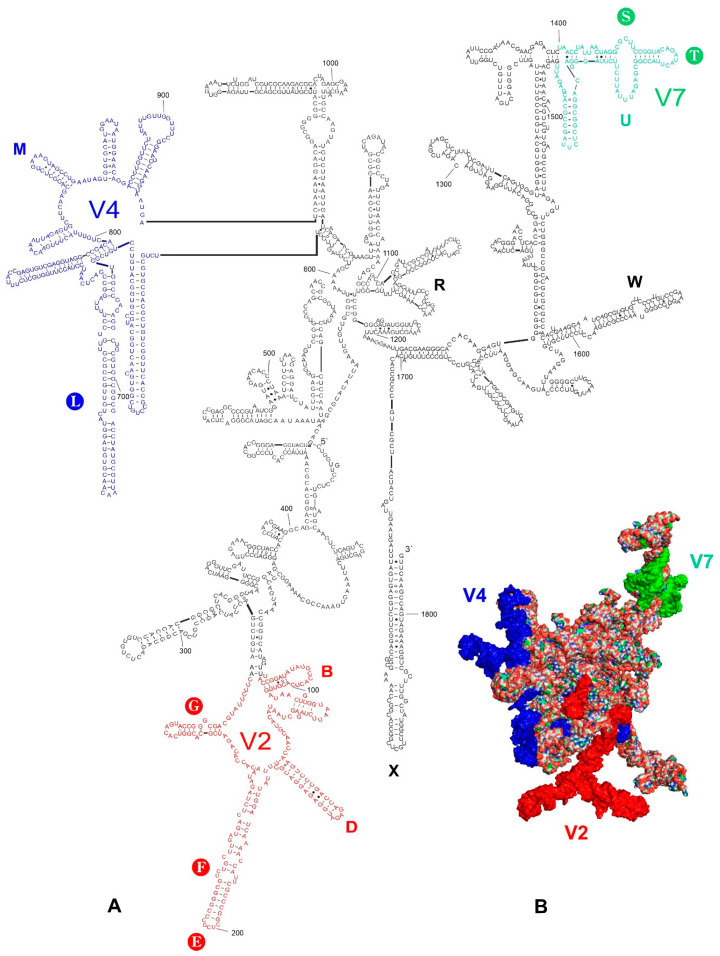
*18S rRNA* of *Thaumastella elizabethae*. (**A**) Secondary structure model. The bases marked in colour represent the hypervariable regions (V2—red, V4—dark blue, V7—green). Thirteen length-variable regions (LVRs) are labelled as capital letters B to W in colours analogous to the base colours representing the hypervariable regions or other sequences’ regions. The capital letters in filled circles indicate six LVRs (E, F, G, L, S, and T) considered to be those that can serve as molecular synapomorphies or autapomorphies in analyses. Base pairing is shown as follows: standard canonical pairs are lines (G–C, A–U), wobble G:U pairs are dots (G·U), A:G or A:C pairs are open circles (A; G, A; C) and other non-canonical pairs are filled circles (e.g., U and U, A and A). The nucleotide numbering follows Wu et al. [20]. (**B**) Tertiary structure model. The fragments marked in colour represent the hypervariable regions (V2—red, V4—dark blue, V7—green).

The secondary and tertiary structure models of *18S rRNA* of *Thaumastella elizabethae* Jacobs, 1989 are provided in Figure 1. The secondary structure models are largely same as these for all other analysed species, with local differences within the certain hypervariable regions (V) and length-variable regions (LVRs) (File S2).

When the base pairing in the sequence of *T. elizabethae* was considered, nucleotides formed 530 pairs (58.6% of all nucleotides in the secondary structure model). The standard canonical pairs (G–C and A–U) were the most common (418 pairs, 78.9%). The wobble G:U pairs were about seven times less commonly formed between paired nucleotides (63 pairs, 11.9%). A:G or A:C pairs and other non-canonical pairs were observed infrequently (18 and 31—or 3.4% and 5.8%—respectively).

The number of nucleotides forming particular types of pairs varied slightly among all studied sequences, i.e., ±1.0–1.5% in the standard canonical pairs, ±1.5–3.2% in the wobble G:U pairs, and ±4.0–7.7% in all other non-canonical pairs. Such a range of variability is consistent with previous data for other Heteroptera species [19,20,21].

### 2.2. Hypervariable Regions (V2, V4, V7) Secondary Structures

The alignment results of the present analyses (File S1) indicate the existence of three hypervariable regions (V2, V4, V7), which is congruent with the results of the previous studies on *18S rRNA* of Heteroptera species [19,20,21]. Predicted secondary hypervariable regions’ structures and their location within the entire gene sequences are shown in Figure 1 for *T*. *elizabethae* and in File S2 for all consensus species.

The sequence length of the hypervariable regions (Table 1) was highly diverse in the V4 region (316–320 nucleotides) and less variable in the V2 and V7 regions (192–194 and 90–91 nucleotides, respectively).

### 2.3. Length-Variable Regions (LVRs) in the Secondary Structures of 18S rRNA

Positions of the length-variable regions (LVRs) within the gene sequences are shown in Figure 1 for *Thaumastella elizabethae* and in File S2 for all analysed consensus species.

The six LVRs (F, M, S, U, R, W) always had a uniform number of nucleotides for each region. This was two nucleotides for LVR F, three for LVR W, four for LVR M and LVR R, five for LVR S and thirteen nucleotides for LVR W (Table 2). All other LVRs showed more or less significant variations in length, but the LVR G appeared to be the most specific because it was detected only in the Thaumastellidae. In species of this family, this LVR included a single nucleotide, whereas in all other taxa, this LVR was absent (Table 2, File S2).

Most notable was the LVR L, which was the longest and the most variable in length among all 13 recognised LVRs (Table 2). Therefore, it was subdivided into subregions to compare its homologous fragments in all analysed sequences. This subdivision was based on the alignment (File S1) and results of the secondary structure predictions (File S2), and the identified subregions were subjected to further analyses.

### 2.4. Length-Variable Region L (LVR L) Secondary Structure

The total length of the LVR L in the studied species varied from 71 to 81 nucleotides (Table 2). It was most variable in the subfamily Cydninae of the Cydnidae (73–81 nucleotides), the outgroup species (72–78 nucleotides) and the species of Parastrachiidae (73–78). The range of variability in other ingroup families showed no differentiation or was insignificant (up to four nucleotides only).

**Figure 2:**
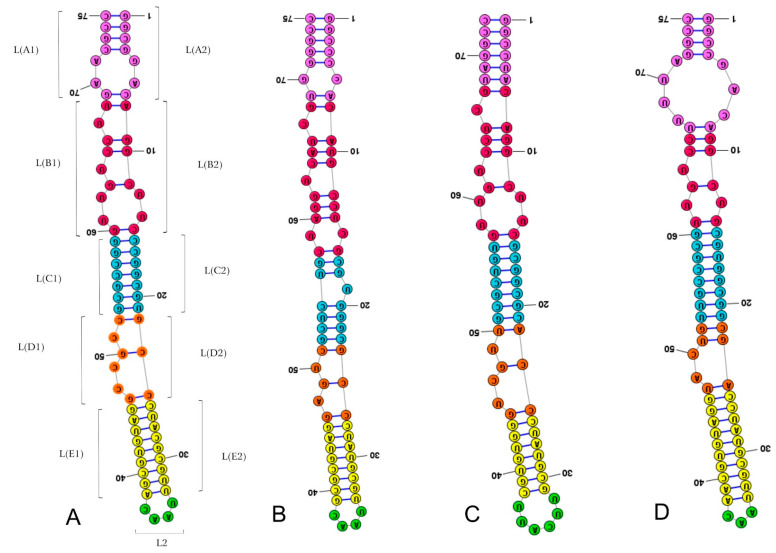
Secondary structure models of the length-variable region L. (**A**) *Riptortus pedestris* (outgroup). (**B**) *Lestonia haustorifera* (Lestoniidae). (**C**) *Parastrachia japonensis* (Parastrachiidae). (**D**) *Thaumastella elizabethae* (Thaumastellidae). Specific subregion bases are marked in the same colour.

The general scheme for the outgroup and three ingroup consensus species, including *Thaumastella elizabethae*, is presented in Figure 2. The subdivision of LVR L into subregions was based on the alignment and results of the secondary structure predictions. These subregions were compared for all consensus species analysed (File S3). The number of nucleotides for each subregion resulting from the comparative analysis is provided in Table 3.

The LB region (LB1 + B2) was the most variable: its number of nucleotides varied from 11 to 20. Two regions (LA and LE) were less variable (in a range of seven nucleotides of variability), while two (LD and L2) were the least variable (in a range of only four nucleotides of variability).

### 2.5. 18S rRNA Tertiary Structure Models

**Figure 3:**
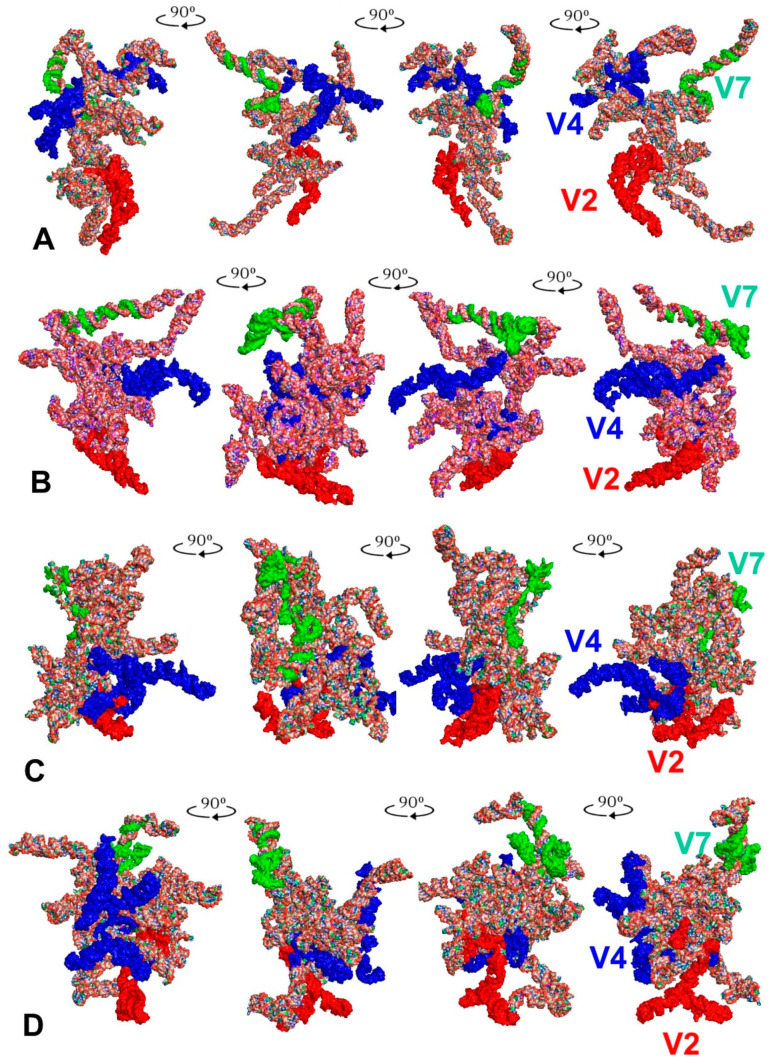
Tertiary structure models of the *18S rRNA* gene. (**A**) *Riptortus pedestris* (outgroup). (**B**) *Parastrachia japonensis* (Parastrachiidae). (**C**) *Lestonia haustorifera* (Lestoniidae). (**D**) *Thaumastella elizabethae* (Thaumastellidae). The hypervariable regions are marked in red (V2), dark blue (V4) and green (V7).

The tertiary structure models of the *18S rRNA* gene were predicted for the 15 consensus species listed in Table 1. The predicted models are shown in Figure 3 for *Riptortus pedestris* (outgroup), *Parastrachia japonensis* (Parastrachiidae), *Lestonia haustorifera* (Lestoniidae) and *Thaumastella elizabethae* (Thaumastellidae) and in File S4 for all consensus species.

**Figure 4:**
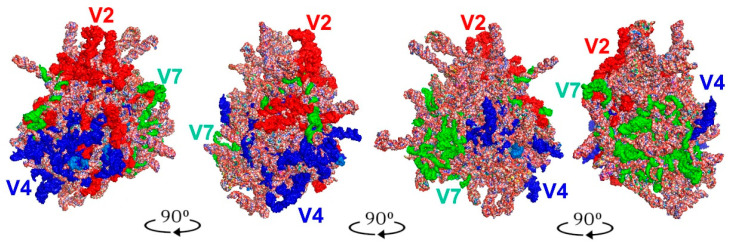
Combined tertiary structure models of the *18S rRNA* gene in 15 analysed consensus species. All sequences were aligned to the outgroup (*Riptortus pedestris*) sequence. The hypervariable regions are marked in red (V2), dark blue (V4) and green (V7).

**Figure 5:**
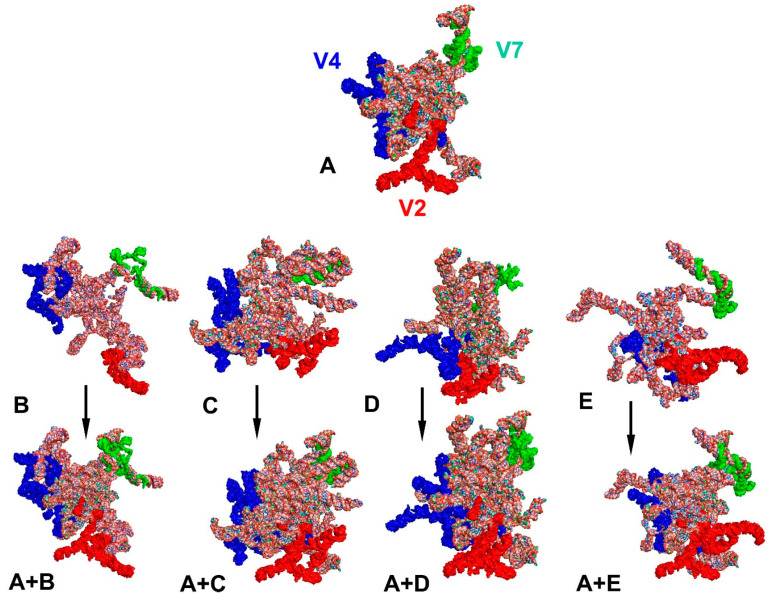
Tertiary structure models of the *18S rRNA* gene in sole and combined aligned views. (**A**) *Thaumastella elizabethae* (Thaumastellidae). (**B**) *Cantao ocellatus* (Scutelleridae). (**C**) *Elasmostethus interstinctus* (Acanthosomatidae). (**D**) *Lestonia haustorifera* (Lestoniidae). (**E**) *Urochela luteovaria* (Urostylididae). The hypervariable regions are marked in red (V2), dark blue (V4) and green (V7).

When all 15 tertiary structures were aligned (Figure 4), their general shape appeared to be very similar, especially in five species. These include *Cantao ocellatus* (Scutelleridae), *Elasmostethus interstinctus* (Acanthosomatidae), *L. haustorifera* (Lestoniidae), *Urochela luteovaria* (Urostylididae) and *T*. *elizabethae* of the family Thaumastellidae, whose systematic position is the object of these considerations. This similarity in their tertiary structures related primarily to the location of the hypervariable regions V2, V4 and V7 (Figure 5). Nevertheless, this correlation regarding the hypervariable regions’ positions was not detected in the other ten species (File S5).

### 2.6. Length-Variable Region L (LVR L) Tertiary Structure

**Figure 6:**
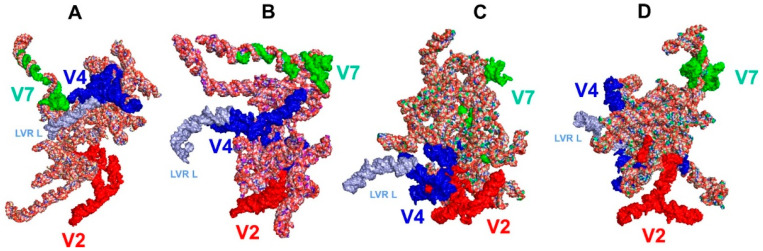
The LVR L position (marked in light blue) within the hypervariable region V4 in the tertiary structure models of the *18S rRNA* gene. (**A**) *Riptortus pedestris* (outgroup). (**B**) *Parastrachia japonensis* (Parastrachiidae). (**C**) *Lestonia haustorifera* (Lestoniidae). (**D**) *Thaumastella elizabethae* (Thaumastellidae). The hypervariable regions are marked in red (V2), dark blue (V4) and green (V7). All sequences are aligned to the outgroup (*R. pedestris*) sequence.

The position of the length-variable region L (LVR L) in the gene tertiary structure for the outgroup (*Riptortus pedestris*) and three ingroup species is shown in Figure 6. The LVR L—the longest part (71 to 81 nucleotides) of the entire hypervariable region V4—is always well recognisable (Figure 6, File S6).

**Figure 7:**
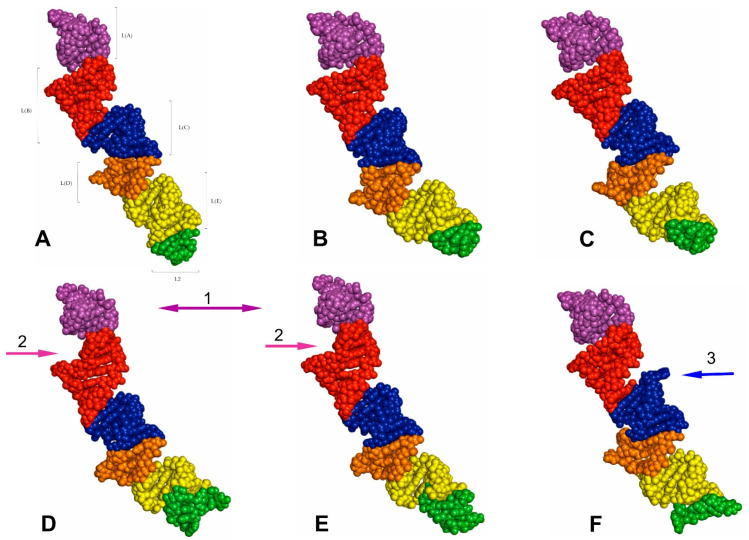
The predicted tertiary structure models of the LVR L. (**A**) *Riptortus pedestris* (outgroup). (**B**) *Canopus* sp. (Canopidae). (**C**) *Cantao ocellatus* (Scutelleridae). (**D**) *Adomerus biguttatus* (Cydnidae: Sehirinae). (**E**) *Parastrachia japonensis* (Parastrachiidae). (**F**) *Fromundus pygmaeus* (Cydnidae: Cydninae). The arrows corresponding in colour to the particular LVR L subregion indicate the fragments that can serve as potential morpho-molecular derived characters: (1, 2) synapomorphies, (3) autapomorphy. Sequences are aligned to the outgroup (*R. pedestris*) sequence.

**Figure 8:**
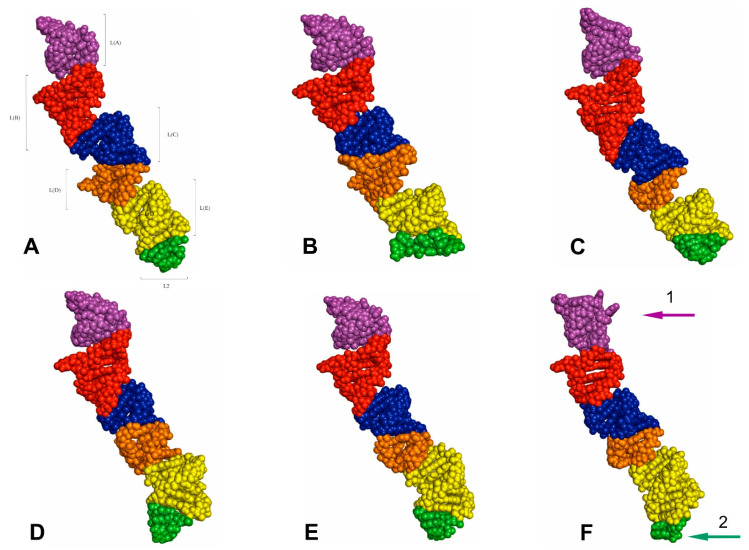
The predicted tertiary structure models of the LVR L. (**A**) *Riptortus pedestris* (outgroup). (**B**) *Eurostus validus* (Tessaratomidae). (**C**) *Lestonia haustorifera* (Lestoniidae). (**D**) *Megymenum* sp. (Megymeninae). (**E**) *Urochela luteovaria* (Urostylididae). (**F**) *Thaumastella elizabethae* (Thaumastellidae). The arrows corresponding in colour to the particular LVR L subregion indicate the fragments that can serve as potential morpho-molecular autapomorphies (1, 2). Sequences are aligned to the outgroup (*R. pedestris*) sequence.

**Figure 9:**
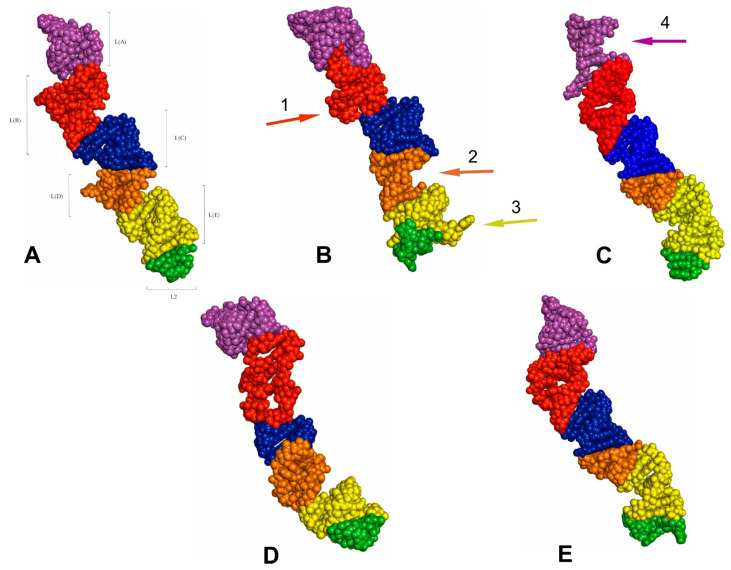
The predicted tertiary structure models of the LVR L. (**A**) *Riptortus pedestris* (outgroup). (**B**) *Coptosoma scutellatum* (Plataspidae). (**C**) *Elasmostethus interstinctus* (Acanthosomatidae). (**D**) *Eurydema maracandica* (Pentatomidae). (**E**) *Thyreocoris scarabaeoides* (Thyreocoridae). The arrows corresponding in colour to the particular LVR L subregion indicate the fragments that can serve as potential morpho-molecular autapomorphies (1–4). Sequences are aligned to the outgroup (*R. pedestris*) sequence.

**Figure 10:**
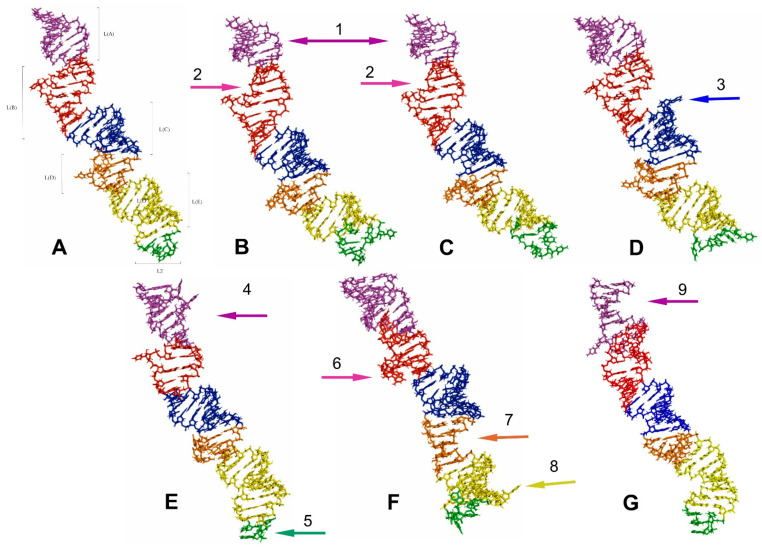
The predicted tertiary structure models of the LVR L, showing ring structures and hydrogen bonds. (**A**) *Riptortus pedestris* (outgroup). (**B**) *Adomerus biguttatus* (Cydnidae: Sehirinae). (**C**) *Parastrachia japonensis* (Parastrachiidae). (**D**) *Fromundus pygmaeus* (Cydnidae: Cydninae). (**E**) *Thaumastella elizabethae* (Thaumastellidae). (**F**) *Coptosoma scutellatum* (Plataspidae). (**G**) *Elasmostethus interstinctus* (Acanthosomatidae). The arrows corresponding in colour to the particular LVR L subregion indicate the fragments that can serve as potential morpho-molecular synapomorphies (1, 2) and autapomorphies (3–9). All sequences are aligned to the outgroup (*R. pedestris*) sequence.

The tertiary structures of the LVR L predicted for all fifteen analysed consensus species are presented in Figure 7, Figure 8, Figure 9 and Figure 10. The fragments that can serve as potential morpho-molecular synapomorphies or autapomorphies are indicated by the arrows corresponding in colour to the particular LVR L subregion (Figure 7, Figure 8, Figure 9 and Figure 10).

## 3. Discussion

### 3.1. The Predicted 18S rRNA Secondary Structures of Pentatomoidea

Comparisons of the *18S rRNA* secondary structure models predicted for 15 pentatomoid consensus species show that they are almost identical. The differences found relate to the limited variations within certain hypervariable regions (V) and significant modifications within the length-variable regions (LVRs).

Among the analysed LVRs, LVR L was the most variable, which is consistent with the results of previous studies [19,20]. The unique attributes of its subregions make them appropriate to serve as apomorphies (autapomorphies or synapomorphies) for a particular taxon or groups of taxa.

### 3.2. Potential Synapomorphies and Apomorphies in LVR L Secondary Structures

The results of the comparative analyses of LVR L secondary structures showed the presence of several synapomorphies and autapomorphies relating to the number of nucleotides in specific subregions (Table 3).

As defined by their nucleotide numbers, the plesiomorphic conditions of the outgroup subregions were frequently shared by the ingroup taxa. The ancestral number of nucleotides for the exact subregion is observed from seven to ten times (for the L2 subregion—tenfold; L(A)–ninefold; L(B) and L(D)—eightfold; and L(C)—sevenfold). The L(E) subregion was the most derived, and demonstrated only apomorphies; its plesiomorphic state of the outgroup was never recovered in the ingroup taxa (Table 3).

Additionally, several synapomorphies were detected for specific ingroup taxa. The presence of six nucleotides in the L2 subregion and fourteen nucleotides in the L(E) subregion can be regarded as synapomorphies for the subfamily Sehirinae of the Cydnidae and for two other families, namely Parastrachiidae and Thyreocoridae. Moreover, synapomorphies for the Sehirinae and the Parastrachiidae were also identified in the L(A) and L(B) subregions (12 and 18 nucleotides, respectively). Two synapomorphies for three families, i.e., Dinidoridae, Tessaratomidae and Pentatomidae, were recovered in the L(C) and L(D) subregions (12 and 11 nucleotides, respectively).

Among all analysed taxa, two families, namely the Thaumastellidae and the Plataspidae, had the highest number of autapomorphies (four). The family Lestoniidae had two autapomorphies, while one autapomorphy was indicated for each of the three following taxa: the Acanthosomatidae, the Thyreocoridae and the subfamily Cydninae of the Cydnidae (Table 3).

Besides its autapomorphies, the Thaumastellidae had one synapomorphy with the Lestoniidae (eight nucleotides in the L(D) subregion) and one with the Plataspidae (16 nucleotides in the L(C) subregion).

However, the results of the present analyses indicate no support for the synapomorphies in LVR L for Lestoniidae + Acanthosomatidae, as previously suggested [20].

### 3.3. The Predicted 18S rRNA Tertiary Structures of Pentatomoidea

Although the secondary structures of all analysed species were almost identical, their predicted tertiary structures varied, sometimes significantly. Only five species—representing Scutelleridae (*C*. *ocellatus*), Acanthosomatidae (*E*. *interstinctus*), Lestoniidae (*L. haustorifera*), Urostylididae (*U*. *luteovaria*) and Thaumastellidae (*T*. *elizabethae*)—had a largely similar overall shape, especially when the location of the hypervariable regions V2, V4 and V7 was considered. This correlation was not detected in the other ten species, since their variable regions (V2, V4, V7) were situated in various locations of their tertiary structures. These results show that the probable *in vivo* tertiary configurations of the *18S rRNAs* in pentatomoid true bugs are not predictable using existing software (3dRNA v2.0 Web Server).

Moreover, this may also result from small nucleolar RNAs (snoRNAs) activities, which can stabilise highly conserved ribosomal RNA secondary structures and affect changes in their tertiary structure [56,57,58,59,60]. However, such modifications have been sporadically analysed in invertebrates [57,59,60]. These cases concern, in particular, the model organisms (mainly nematodes and *Drosophila* species), but to my knowledge, never any other insects. It will be essential to verify in the future whether any of these rRNA modifications caused by snoRNAs could change the tertiary structures of length-variable *18S rRNA* regions not only in Hemiptera but also in the entire Hemimetabola.

### 3.4. Potential Synapomorphies and Autapomorphies in LVR L Tertiary Structure

The results show only two analysed consensus species, *Adomerus biguttatus* (Cydnidae: Sehirinae) and *Parastrachia japonensis* (Parastrachiidae), share strict morpho-molecular synapomorphies in the LVR L structure. These two synapomorphies correspond to two subregions: L(A) and L(B) (Figure 7D,E and Figure 10B,C).

Moreover, four consensus species—*Coptosoma scutellatum* (Plataspidae), *Thaumastella elizabethae* (Thaumastellidae), *Fromundus pygmaeus* (Cydnidae: Cydninae) and *Elasmostethus interstinctus* (Acanthosomatidae)—exhibit the morpho-molecular autapomorphies in the tertiary structure of their LVR L (Figure 7, Figure 8, Figure 9 and Figure 10).

In the *C*. *scutellatum* LVR L tertiary structure, three autapomorphies were identified, namely in L(B), L(D) and L(E) (Figure 9B and Figure 10F). The *T. elizabethae* LVR L tertiary structure revealed two autapomorphies, one in L(A) and one in L2 (Figure 8F and Figure 10E). Moreover, two species had only one morpho-molecular autapomorphy. These were *F*. *pygmaeus*, exhibiting a single morpho-molecular autapomorphy in the L(C) subregion (Figure 7F and Figure 10D), and *E*. *interstinctus*, with a single autapomorphy in the L(A) subregion (Figure 9C and Figure 10G).

All other consensus species had no well-defined morpho-molecular-derived characters (neither synapomorphies nor autapomorphies) in their predicted tertiary *18S rRNA* structures.

### 3.5. Systematic Position of Thaumastellidae within Pentatomoidea

The results of the present comparative analyses of the predicted *18S rRNA* secondary and tertiary structures—in addition to the morphological [22,23,24,25,33], biochemical [32] and phylogenetic evidence to date [7,20,35,36,40]—support the hypothesis that the Thaumastellidae are phylogenetically distinct from the Cydnidae and should not be placed within the latter as a subfamily.

Thus far, the only morphological character considered a synapomorphy for all taxa within the broadly conceived Cydnidae (‘cydnoid complex’ sensu Lis et al. [7]; Cydnidae sensu Schuh and Weirauch [27]) was the presence of the coxal combs, unique within Heteroptera [27,43]. However, evidence for these structures’ independent origin has recently been provided [7], indicating the Thaumastellidae as the out-group for all other families of Pentatomoidea as suggested thus far [20,32,33,34,35,36].

As was already demonstrated [20]—and confirmed in the present study—the Thaumastellidae have a single nucleotide in the LVR G, which is unique among Pentatomoidea. The presence of this nucleotide should be regarded as the family autapomorphy [20]. All other pentatomoidean taxa (including the Cydnidae) display the plesiomorphic character and have no nucleotide in the LVR G [20] (present study).

Moreover, the current analyses have shown for the first time that the Thaumastellidae hold four morpho-molecular autapomorphies in the secondary structure of LVR L subregions L2, LA, LB and LE (Table 3), two of which (in the L2 and LA subregions) are also detectable in the tertiary structure (Figure 8 and Figure 10). Other autapomorphies in the number of the nucleotides in LVR L subregion secondary structures (one in the Plataspidae LC subregion and the second in the Lestoniidae LD subregion) (Table 3) are, unfortunately, not reflected in the tertiary structures (Figure 7, Figure 8, Figure 9 and Figure 10).

Furthermore—and most importantly—the Thaumastellidae did not share any synapomorphies with any of the two Cydnidae subfamilies (Cydninae and Sehirinae), in which the Thaumastellidae have previously been classified based on morphological characters [20,27,34,46,47,48,49].

Irrespective of the findings of the present analyses, the hypothesis of the sister relationship of Thaumastellidae to all other Pentatomoidea is strongly supported by several other characters. These include the presence of an m-chromosome, unknown elsewhere in this superfamily [31,32]; the chemical composition of the scent gland secretions, which are intermediate between Lygaeoidea and Pentatomoidea [32]; and the structure of the spermatheca, which is more lygaeoid or pyrrhocoroid than pentatomoid [33].

Therefore, it is suggested here to retain the Thaumastellidae as an independent family within the superfamily Pentatomoidea, not a part of the family Cydnidae (regardless of its internal classification adopted).

### 3.6. Other Families’ Relationships within the Superfamily Pentatomoidea

The current analyses identified two significant relationships among the investigated pentatomoid families at the molecular level.

The first concerns the synapomorphies in the LVR L subregion secondary structure of the subfamily Sehirinae (Cydnidae), the Parastrachiidae and the Thyreocoridae (Table 3). These contrasted with the predicted tertiary structures, which indicated that only Sehirinae and Parastrachiidae (but not Thyreocoridae) shared morpho-molecular synapomorphies in the LVR L subregions. The very close sister affinities of the two taxa—i.e., Sehirinae and Parastrachiidae—have been suggested previously based on phylogenetic analyses, where they always formed a highly supported monophylum [7,36,44]. This finding is also supported by the results of existing morphological analyses [33,40,61,62,63,64,65,66] and by similarities in the oviposition mode and maternal care habits of the taxa [67,68,69,70,71,72,73]. The results of the present analyses appear to support the close relationship between the subfamily Sehirinae (of the Cydnidae) and the family Parastrachiidae. However, the current analyses did not confirm the close affinity between the Parastrachiidae and the Thyreocoridae, as suggested thus far [40,44].

Moreover, two synapomorphies in LVR L secondary structures were revealed for three other families: Dinidoridae, Tessaratomidae and Pentatomidae. Although the Dini-doridae and Tessaratomidae have sometimes been considered sister taxa in phylogenetic analyses [9,10,40,74,75], their predicted LVR L tertiary structures did not demonstrate any morpho-molecular synapomorphies. Therefore, the present study cannot convincingly support a close relationship between Dinidoridae and Tessaratomidae.

All other consensus species had no distinct morpho-molecular synapomorphic characters in their *18S rRNA* secondary and tertiary structures.

## 4. Materials and Methods

### 4.1. Reconstruction of 18S rRNA Secondary Structure Models

The secondary structure of the *18S rRNA* for each analysed taxon was designed according to the gene’s universal model provided for insects [3]. Some modifications proposed for Heteroptera [19,20] were considered, and the fragments containing the length-variable regions (LVRs) were reinterpreted for each sequence using RNAstructure ver. 6.3 [76]. To avoid misinterpretations of the nucleotide substitutions during *18S rRNA* secondary structure reconstruction—particularly in the regions containing LVRs [20]—the compensatory or semi-compensatory substitutions [77] were examined. This allowed for the delimitation of the variable regions and conservative portions of this gene sequence. The optimal model was selected with the principle of co-variation [78] and hypervariable region numbering, following that provided by Neefs et al. [14], Gillespie et al. [2], Yu et al. [19] and Wu et al. [20]. The numbering system for the length-variable regions (LVRs) in the present study was adopted from Neefs et al. [14], Xie et al. [3] and Wu et al. [20]. The nucleotide numbering of the entire gene sequences follows Wu et al. [20].

### 4.2. Selection of the Hypervariable Regions (V) and Length-Variable Regions (LVRs) for Analyses

Nine hypervariable regions (V1–V9) were recognised in the eukaryotic *18S rRNA* model [14]. However, only three (V2, V4, V7) were indicated [2,3,50] to be fast-evolving and to significantly impact the phylogenetic reconstructions of relationships among high-level taxa in Hexapoda. As far as the length-variable regions (LVRs) are concerned, 24 such regions (A to X) within the nine domains (V1–V9) were recognised [3]. Most of these (18 out of 24) are restricted to the three hypervariable domains (V2, V4, V7), and the length of various LVRs can be order-specific [3]. Nevertheless, results concerning Heteroptera [19,20] have suggested that the local length sequence variations are restricted only to thirteen separate LVRs: five (B, D–G) in the V2 hypervariable region, two (L–M) in the V4 region, three (S–U) in the V7 region and three (R, X, W) in other parts of the sequences (Figure 1). However, only three LVRs (E, F, G) in the V2 region, one LVR (L) in the V4 region and two LVRs (S, T) in the V7 region have been identified as those that can potentially serve as morpho-molecular synapomorphies (or autapomorphies) for taxa within the superfamily Pentatomoidea [19,20]. Therefore, only the six LVRs mentioned above (E, F, G, L, S, T) were considered in the analyses (Figure 1).

### 4.3. Prediction of LVR Secondary Structures

Secondary structures of the selected length-variable regions were predicted with the computer program RNAstructure ver. 6.3 [76]. A three-step procedure was applied to the comparative sequence analysis in order to improve the quality of the predicted final structure. Firstly, the secondary structures of LVRs were predicted separately for each species. The analyses included calculating a partition function, predicting a minimum free energy (MFE) structure, finding structures with maximum expected accuracy and predicting pseudoknots [76,78]. Then, a secondary structure common to the two sequences was predicted [76] for five pentatomoid families, each represented by only two species in the analyses (Dinidoridae, Parastrachiidae, Plataspidae, Scutelleridae and Thaumastellidae). Finally, secondary structures common to three or more sequences were calculated [76]. This was performed for the outgroup, four pentatomoid families (Acanthosomatidae, Pentatomidae, Thyreocoridae, Urostylididae) and, in particular, for the subfamilies recognised within the family Cydnidae [7], which were all represented by three or more species in the analyses. The analyses covering three or more sequences fold them into their common lowest free energy conformations and combine the capabilities of Multilign and TurboFold to create distinct sets of possible structures for multiple sequences [76]. The structure exhibiting the lowest free energy was selected for further consideration. For this study, the species selected by the computer program RNAstructure ver. 6.3 [76] as exhibiting a secondary structure common to two or more sequences was considered to be the ‘consensus species’ for these sequences (Table 1 and Table S1). Additionally, the LVR L, the longest among all LVRs, was subdivided into subregions to compare its homologous fragments in analysed sequences; this subdivision was based on the alignment and results of the secondary structure predictions.

### 4.4. Prediction of Tertiary Structures

3dRNA v2.0 Web Server (http://biophy.hust.edu.cn/new/3dRNA, accessed on 15 January 2022) was used for the entire *18S rRNA* gene tertiary structure prediction [79]. This fast, automatic method with high-accuracy RNA tertiary structure prediction uses sequence and secondary structure information to build three-dimensional RNA structures [79]. Moreover, 3dRNA Web Server can predict relatively long RNAs’ tertiary structures, even those exceeding 1400 nucleotides [79]. When constructing the secondary structures for the various *18S rRNAs* and predicting the secondary structures for the LVRs, pseudoknot presence or absence was considered [76]. Thus, the possible occurrence of pseudoknots in the tertiary structures was also taken into account when the entire *18S rRNA* gene was addressed. Therefore, an optimisation procedure and the ‘ProbKnot’ method [80,81,82] were applied in the analyses, which was particularly advantageous for predicting the *18S rRNA* tertiary structures.

RNAComposer (http://rnacomposer.ibch.poznan.pl, accessed on 17 January 2022), a fully automated RNA structure modelling server, was used for LVR tertiary structure prediction [83,84]. RNAComposer’s method allows for the building of RNA structures up to 500 nucleotides in length, including the possible occurrence of pseudoknots with high accuracy [83,84]. In the analyses, twenty 3D RNA models were generated for each LVR sequence, and the best model of the lowest free energy was selected for further examination. All secondary structures were visualised using RNAstructure ver. 6.3 [76], while the tertiary structural images were visualised using PyMol software ver. 2.4.0 [85].

### 4.5. Concept of the Morpho-Molecular Structures Potentially Serving as Derived Characters

The concept of morpho-molecular-derived characters (autapomorphies and synapomorphies) of the nucleotide sequences in the predicted *18S rRNA* secondary structures follows Ouvrard et al. [15], Xie et al. [3] and Yu et al. [19].

Unfortunately, a strategy for identifying morpho-molecular apomorphies in the predicted *18S rRNA* (or any other ribosomal RNA) tertiary structures has not yet been developed. As already suggested [86], the homologous sequences of amino acids or nucleotides should be explored as much as possible in order to discover molecular apomorphic characters.

It is known that similar three-dimensional structural elements usually have similar secondary-structure elements [87]. Therefore, a two-step procedure was proposed for recognising the apomorphies in the predicted LVR tertiary structures of the analysed *18S rRNA* sequences. Firstly, all morpho-molecular LVR tertiary structures based on the extent of uniqueness [88] were identified. Then, those LVR tertiary structures that also had their distinctness confirmed at the level of secondary structures were considered as the derived characters (apomorphies and synapomorphies) and were subjected to further analysis.

### 4.6. Selection of Taxa

The results of a recent molecular analysis on the ‘cydnoid complex’ within Pentatomoidea [7] were selected as the most appropriate starting point for the investigations of the rRNA secondary and tertiary structures. A survey [7] based on the *18S + 28S ribosomal DNA* genes included the largest number of Cydnidae species (including two species of *Thaumastella*) and encompassed all Pentatomoidea families bearing sequences of *18S rDNA* deposited in GenBank. Moreover, the tree resulting from the *18S rDNA* analysis had the same topology as that recovered by the combined *18S + 28S ribosomal DNA* genes [7]. Therefore, the Bayesian analysis tree of the combined *28S + 18S rDNA* dataset using the GTR + G/GTR + G + I substitution model recovered under the Akaike Information Criterion [7] was selected as a base for the analysis (Figure A1B). A list of species used for the present study with GenBank accession numbers for *18S rDNA* [7,10,20,40,52,76,89,90,91] is provided in Table S1.

## 5. Conclusions

1. For the first time among Hexapoda, the tertiary structures of the entire SSU were predicted and used in the analyses of the relationships among taxa, representing the heteropteran superfamily Pentatomoidea.
2. Results show that the probable in vivo configuration of the *18S rRNA* tertiary structure is not predictable using only the secondary structure models and existing software (3dRNA v2.0 Web Server). This may also be the result of small nucleolar RNAs (snoRNAs) that can not only stabilise highly conserved ribosomal RNA secondary structures but can affect changes in their tertiary structure.
3. The number of nucleotides in the hypervariable regions (V2, V4, V7) and the length-variable regions (LVRs) of the *18S rDNAs* show variability, suggesting that the secondary and tertiary structures of *18S rRNAs* could be more diverse than has been thought to date.
4. The results concerning Pentatomoidea suggest that the local length sequence variations in the secondary structure models are restricted only to thirteen separate LVRs. However, only three LVRs (E, F, G) in the V2 region, one LVR (L) in the V4 region and two LVRs (S, T) in the V7 region were identified as those that could potentially serve as morpho-molecular apomorphies (synapomorphies or autapomorphies) for taxa within the superfamily Pentatomoidea.
5. Out of the six LVRs mentioned above, only LVR L appeared to be the most appropriate length-variable region for phylogenetic relationship analyses when considering the secondary and tertiary structure models.
6. The proposal for a new LVR L secondary structure subdivision presented in this study works well for the superfamily Pentatomoidea but should be verified in further studies in other groups within the Heteroptera.
7. It is suggested that the Thaumastellidae should remain as an independent family within the superfamily Pentatomoidea, not a part of the family Cydnidae (regardless of its internal classification adopted).
8. The predicted secondary and tertiary structures indicated a close relationship between Sehirinae and Parastrachiidae, with shared morpho-molecular synapomorphies in the LVR L subregions.
9. It seems crucial to incorporate the methods of rRNA secondary and tertiary structure analyses with phylogenetic evaluations.

## Figures and Tables

**Table 1:** The number of nucleotides of the hypervariable regions V2, V4, and V7 in the *18S rRNA* of the analysed taxa. For the ‘consensus species’ definition, see the ‘Materials and Methods’ section.

Taxon Group	Consensus Species	Number of Nucleotides		
		V2	V4	V7
outgroup	*Riptortus pedestris* (Fabricius, 1775)	192	318	91
Acanthosomatidae	*Elasmostethus interstinctus* (Linnaeus, 1758)	194	317	91
Canopidae	*Canopus* sp.	193	316	91
Cydnidae: Cydninae	*Fromundus pygmaeus* (Dallas, 1851)	193	317	91
Cydnidae: Sehirinae	*Adomerus biguttatus* (Linnaeus, 1758)	194	316	91
Dinidoridae	*Megymenum* sp.	193	316	91
Lestoniidae	*Lestonia haustorifera*	194	318	91
Parastrachiidae	*Parastrachia japonensis* (Scott, 1880)	193	316	90
Pentatomidae	*Eurydema maracandica* (Oshanin, 1871)	193	316	91
Plataspidae	*Coptosoma scutellatum* (Geoffroy, 1785)	193	320	91
Scutelleridae	*Cantao ocellatus* (Thunberg, 1784)	193	316	91
Tessaratomidae	*Eurostus validus* (Dallas, 1851)	193	316	91
Thaumastellidae	*Thaumastella elizabethae* (Jacobs, 1989)	194	318	90
Thyreocoridae	*Thyreocoris scarabaeoides* (Linnaeus, 1758)	194	316	91
Urostylididae	*Urochela luteovaria* (Distant, 1881)	194	316	91

**Table 2:** The nucleotide numbers of the LVRs in the *18S rRNA* of the analysed taxa.

Taxon Group	Number of Nucleotides												
	B	D	E	F	G	L	M	S	T	U	R	W	X
outgroup	10–11	4–5	4–5	2	0	72–78	4	5	8–9	13	4	3	4–6
Acanthosomatidae	11	4–5	5–6	2	0	74–75	4	5	8	13	4	3	5–8
Canopidae	11	4	5	2	0	73	4	5	8	13	4	3	5
Cydnidae: Cydninae	11	4	5	2	0	73–81	4	5	8	13	4	3	5
Cydnidae: Sehirinae	11	4	5	2	0	73–75	4	5	8	13	4	3	5
Dinidoridae	11	4	5	2	0	73	4	5	8	13	4	3	4–5
Lestoniidae	11	4	6	2	0	75	4	5	8	13	4	3	5
Parastrachiidae	11	4	5	2	0	73–78	4	5	8–10	13	4	3	5
Pentatomidae	11	4	5	2	0	72–74	4	5	8	13	4	3	5
Plataspidae	11	4	5	2	0	77	4	5	8	13	4	3	5
Scutelleridae	11	4	5	2	0	73	4	5	8	13	4	3	5
Tessaratomidae	11	4	5	2	0	73	4	5	8	13	4	3	5
Thaumastellidae	11	4	5	2	1	72–75	4	5	8	13	4	3	5
Thyreocoridae	11	4	5	2	0	71–74	4	5	8	13	4	3	5
Urostylididae	11–12	4	5	2	0	73–74	4	5	8–9	13	4	3	5

**Table 3:** The nucleotide numbers of the subregions of the LVR L. The autapomorphies for Thaumastellidae are indicated in red, Plataspidae in purple, Lestoniidae in green, Acanthosomatidae in yellow, Thyreocoridae in grey and Cydnidae (specifically Cydninae) in blue.

Taxon Group	Consensus Species	Total Length	Number of Nucleotides of the LVR L Subregions					
			L2	LA (A1 + A2)	LB (B1 + B2)	LC (C1 + C2)	LD (D1 + D2)	LE (E1 + E2)
outgroup	*R*. *pedestris*	75	4	14 (7 + 7)	16 (9 + 7)	14 (7 + 7)	9 (6 + 3)	18 (9 + 9)
Acanthosomatidae	*E*. *interstinctus*	74	4	15 (7 + 8)	16 (9 + 7)	14 (7 + 7)	9 (6 + 3)	16 (8 + 8)
Canopidae	*Canopus* sp.	73	4	14 (7 + 7)	16 (9 + 7)	14 (7 + 7)	9 (6 + 3)	16 (8 + 8)
Cydnidae: Cydninae	*F*. *pygmaeus*	74	4	14 (7 + 7)	16 (9 + 7)	15 (8 + 7)	9 (6 + 3)	16 (8 + 8)
Cydnidae: Sehirinae	*A*. *biguttatus*	73	6	12 (6 + 6)	18 (10 + 8)	14 (7 + 7)	9 (6 + 3)	14 (7 + 7)
Dinidoridae	*Megymenum* sp.	73	4	14 (7 + 7)	16 (9 + 7)	12 (6 + 6)	11 (7 + 4)	16 (8 + 8)
Lestoniidae	*L*. *haustorifera*	75	4	14 (7 + 7)	20 (11 + 9)	13 (6 + 7)	8 (5 + 3)	16 (8 + 8)
Parastrachiidae	*P*. *japonensis*	73	6	12 (6 + 6)	18 (10 + 8)	14 (7 + 7)	9 (6 + 3)	14 (7 + 7)
Pentatomidae	*E*. *maracandica*	73	4	14 (7 + 7)	16 (9 + 7)	12 (6 + 6)	11 (7 + 4)	16 (8 + 8)
Plataspidae	*C*. *scutellatum*	77	4	18 (9 + 9)	14 (10 + 4)	16 (8 + 8)	10 (5 + 5)	15 (7 + 8)
Scutelleridae	*C*. *ocellatus*	73	4	14 (7 + 7)	16 (9 + 7)	14 (7 + 7)	9 (6 + 3)	16 (8 + 8)
Tessaratomidae	*E*. *validus*	73	4	14 (7 + 7)	16 (9 + 7)	12 (6 + 6)	11 (7 + 4)	16 (8 + 8)
Thaumastellidae	*T*. *elizabethae*	75	3	17 (9 + 8)	11 (6 + 5)	16 (8 + 8)	8 (5 + 3)	20 (10 + 10)
Thyreocoridae	*T*. *scarabaeoides*	74	6	14 (7 + 7)	17 (10 + 7)	14 (7 + 7)	9 (6 + 3)	14 (7 + 7)
Urostylididae	*U*. *luteovaria*	73	4	14 (7 + 7)	16 (9 + 7)	14 (7 + 7)	9 (6 + 3)	16 (8 + 8)

## Data Availability

Not applicable.

## Supplementary Materials

The supporting information can be downloaded at: https://www.mdpi.com/article/10.3390/ijms24097758/s1.
