# Supplementary material for: Molecular Apomorphies in the Secondary and Tertiary Structures of Length-Variable Regions (LVRs) of 18S rRNA Shed Light on the Systematic Position of the Family Thaumastellidae (Hemiptera: Heteroptera: Pentatomoidea)
Source: Int J Mol Sci. 2023 Apr 24;24(9):7758. doi: 10.3390/ijms24097758 (PMC10178826; doi:10.3390/ijms24097758)
Supplement: Supplementary file 1 [file ijms-24-07758-s001.zip › FILE S2.pdf]

File S2

The predicted secondary structure models of the 18S rRNA gene for 15 analysed consensus species; for the explanation, see Figure 2.

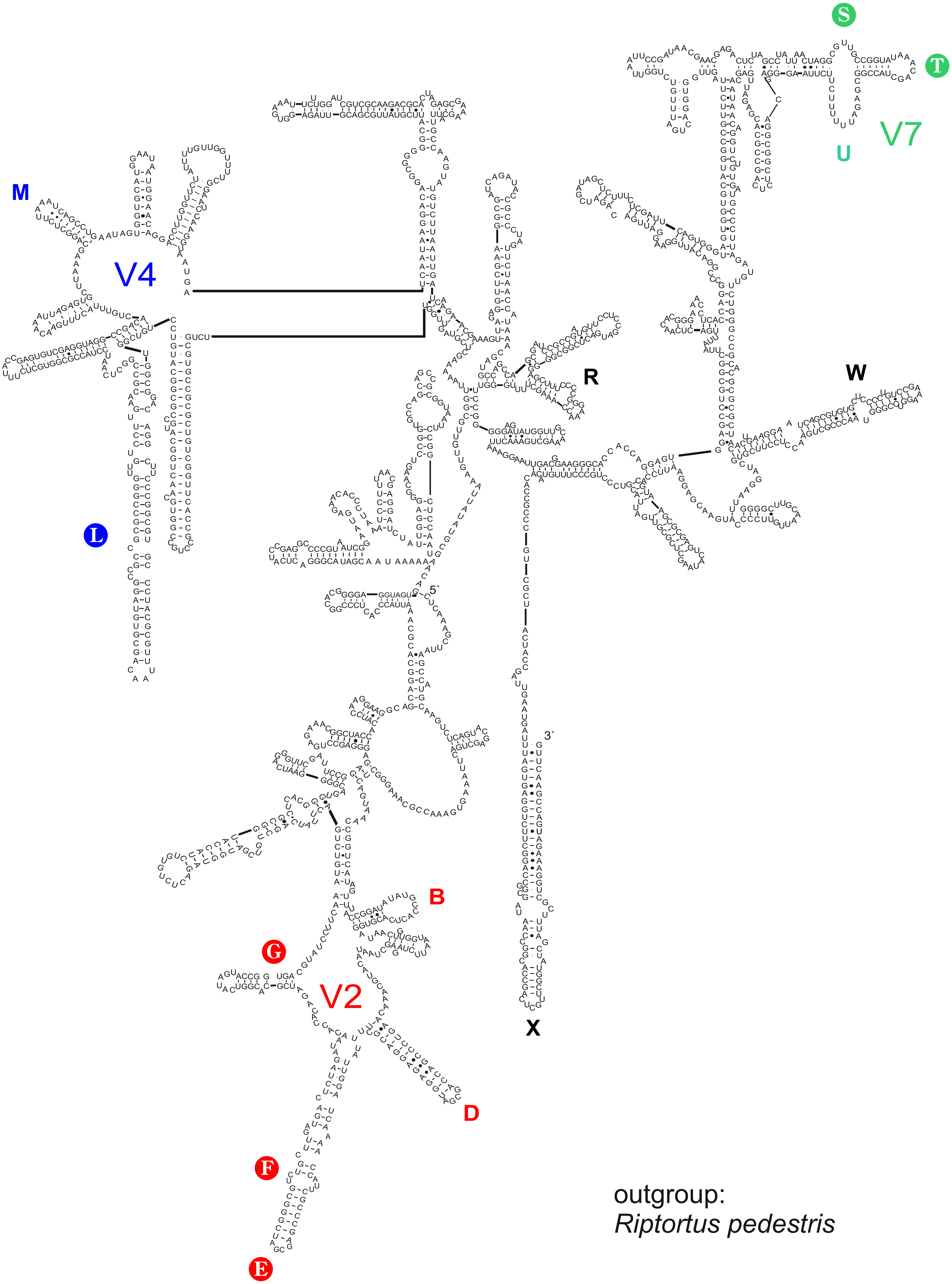

outgroup:
Riptortus pedestris

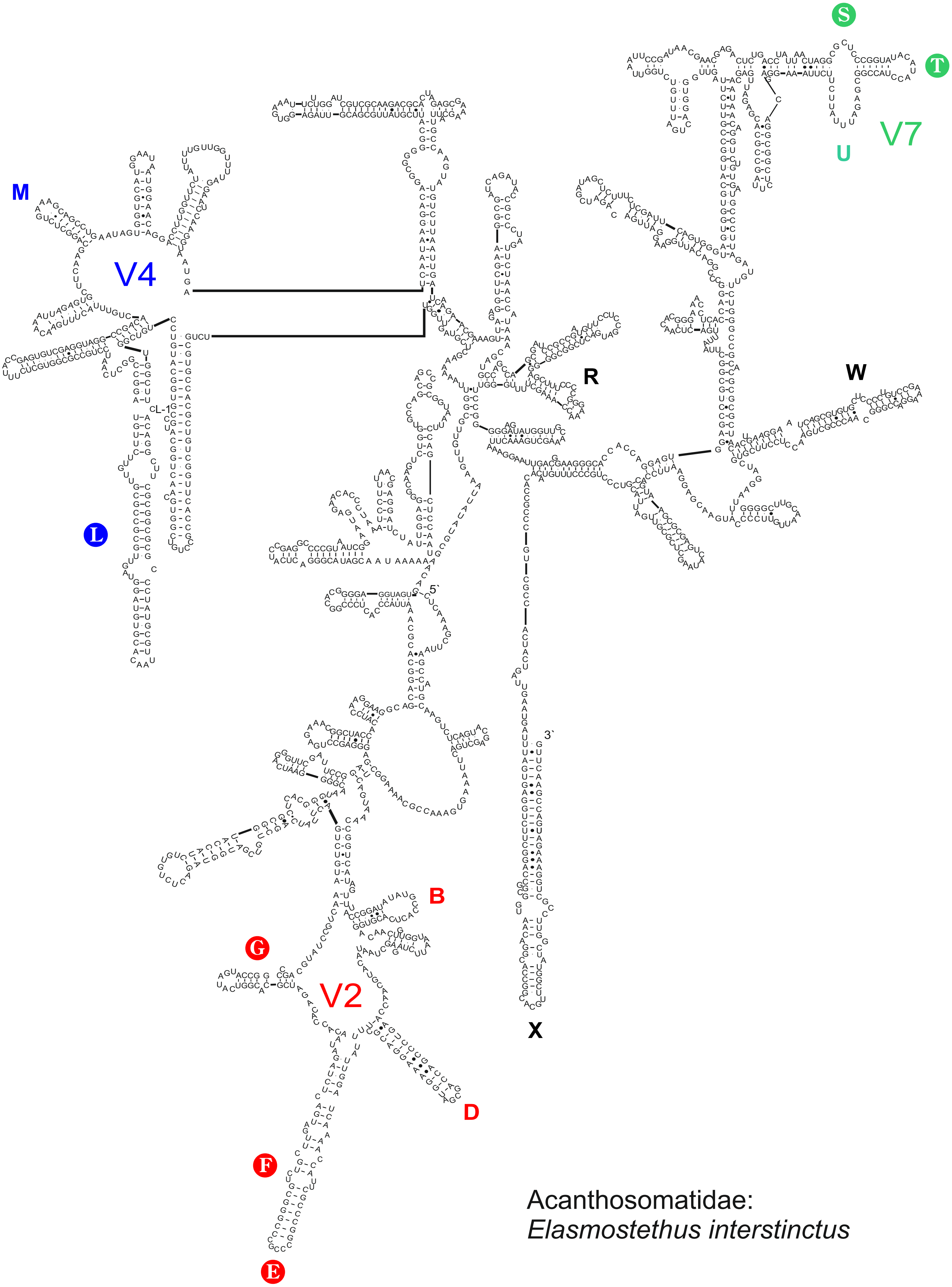

Acanthosomatidae:
Elasmostethus interstinctus

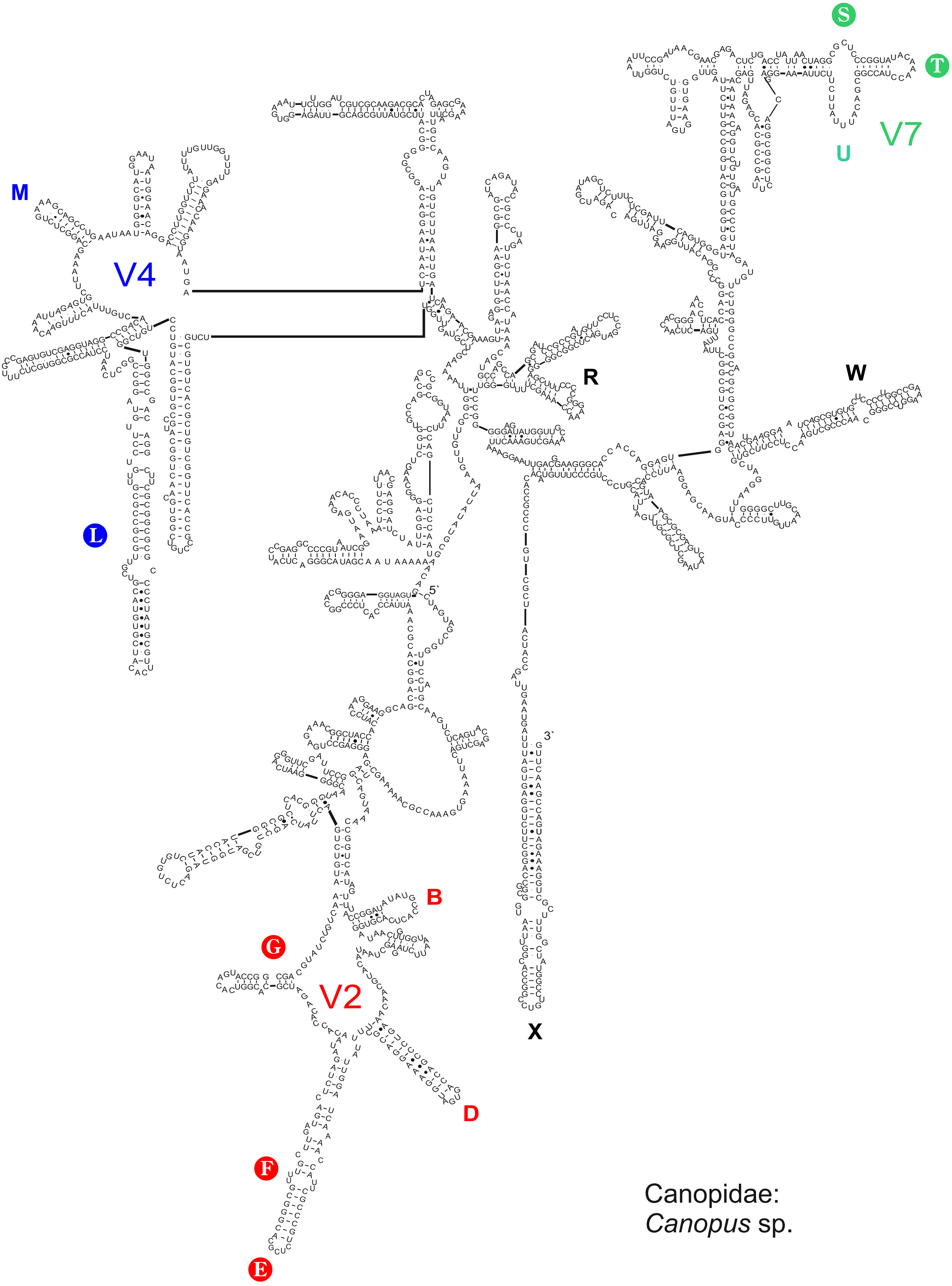

Canopidae:
Canopus sp.

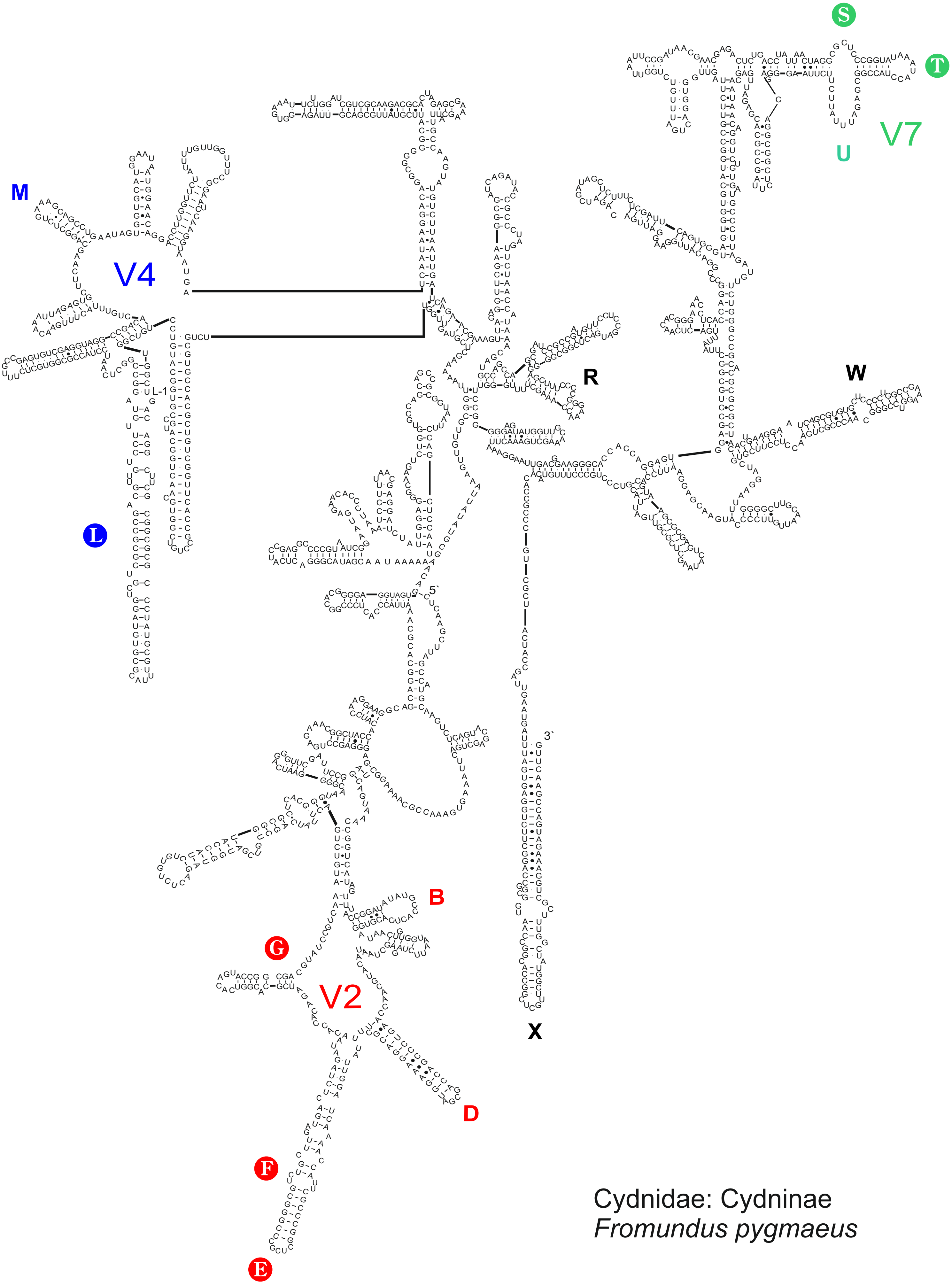

Cydnidae: Cydninae
Fromundus pygmaeus

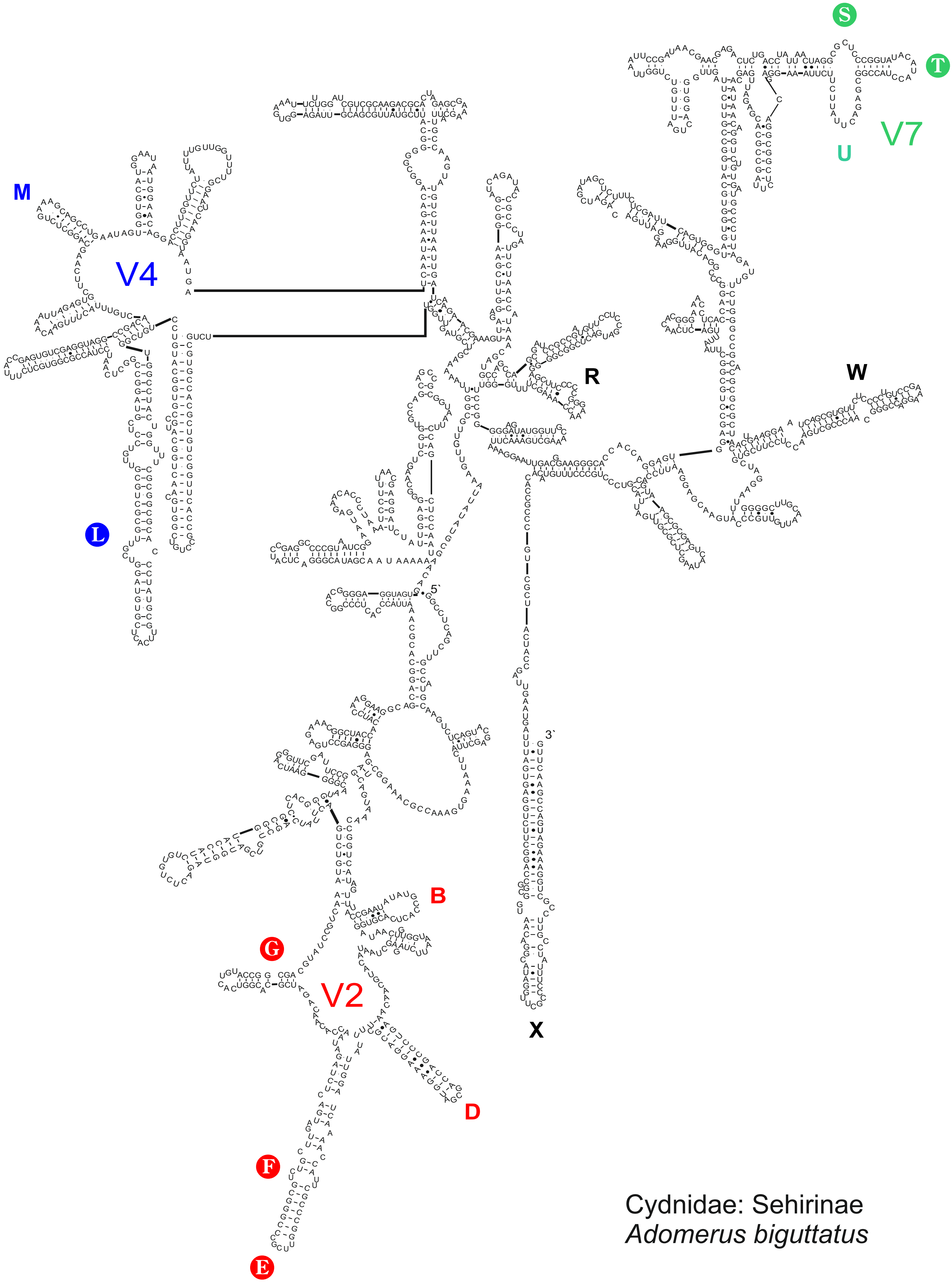

M
V4
L
S
T
V7
U
R
W
X
5`
3`
B
G
V2
D
F
E
Cydnidae: Sehirinae
Adomerus biguttatus

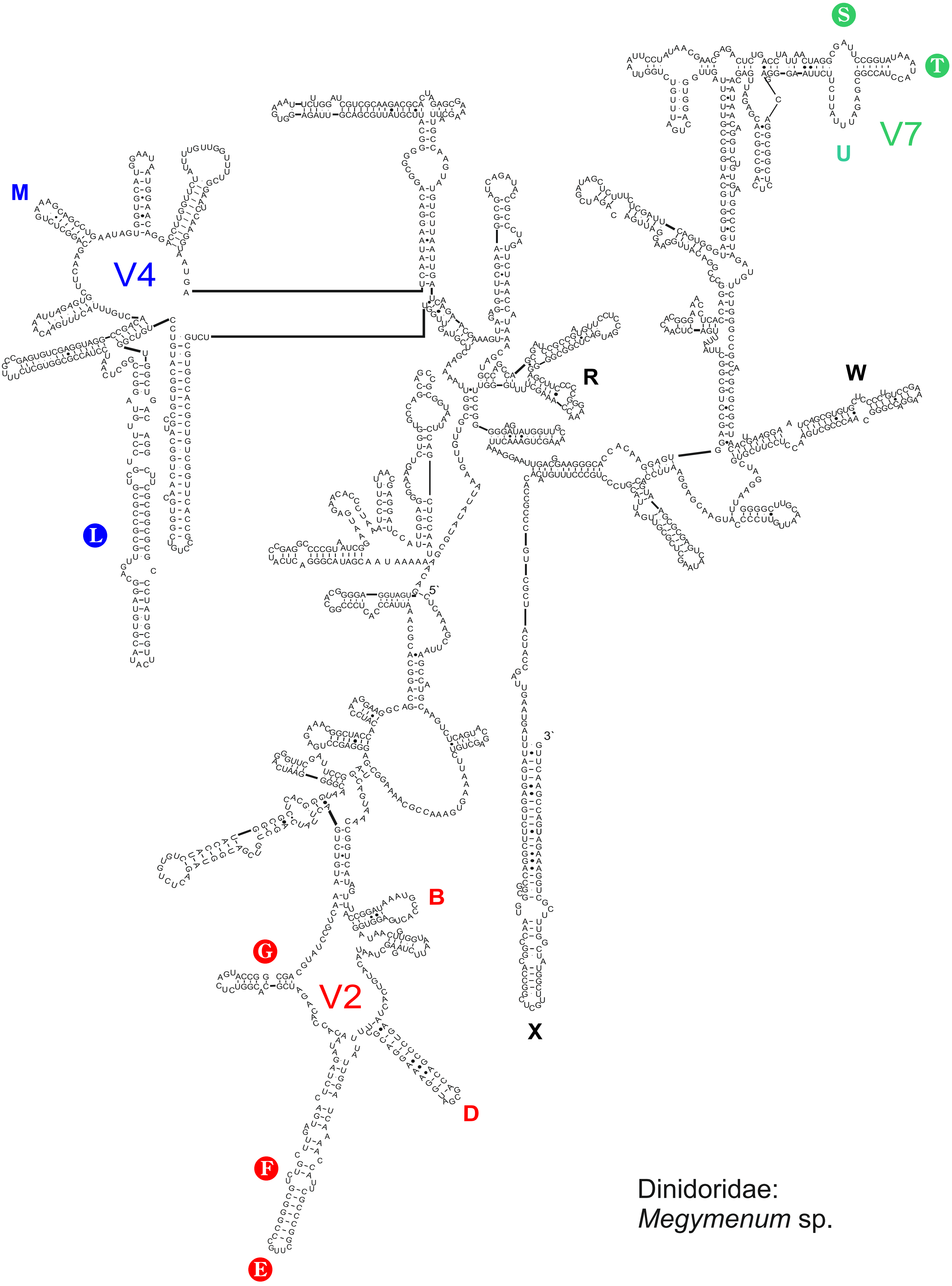

Dinidoridae:
Megymenum sp.

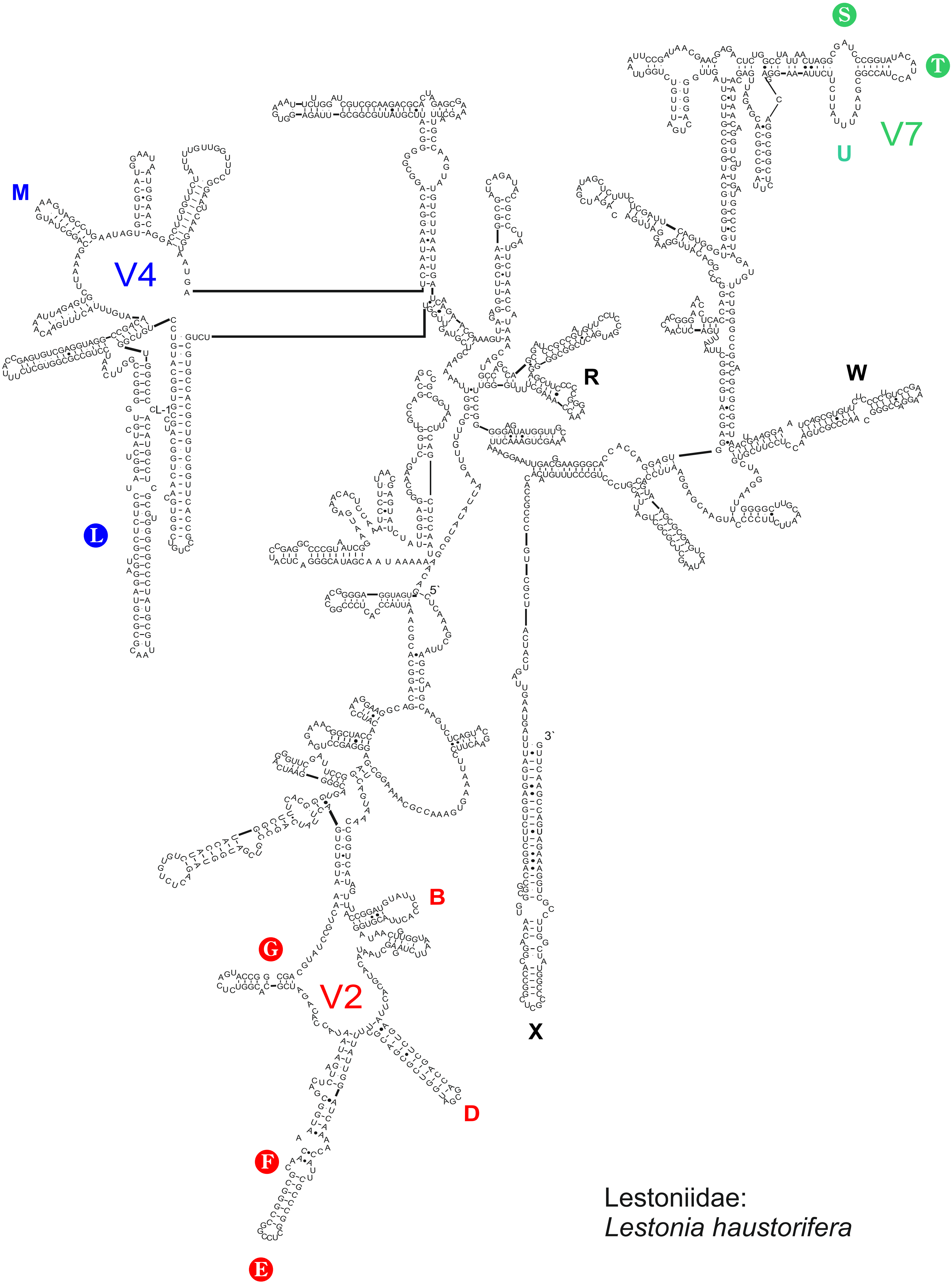

Lestoniidae:
Lestonia haustorifera

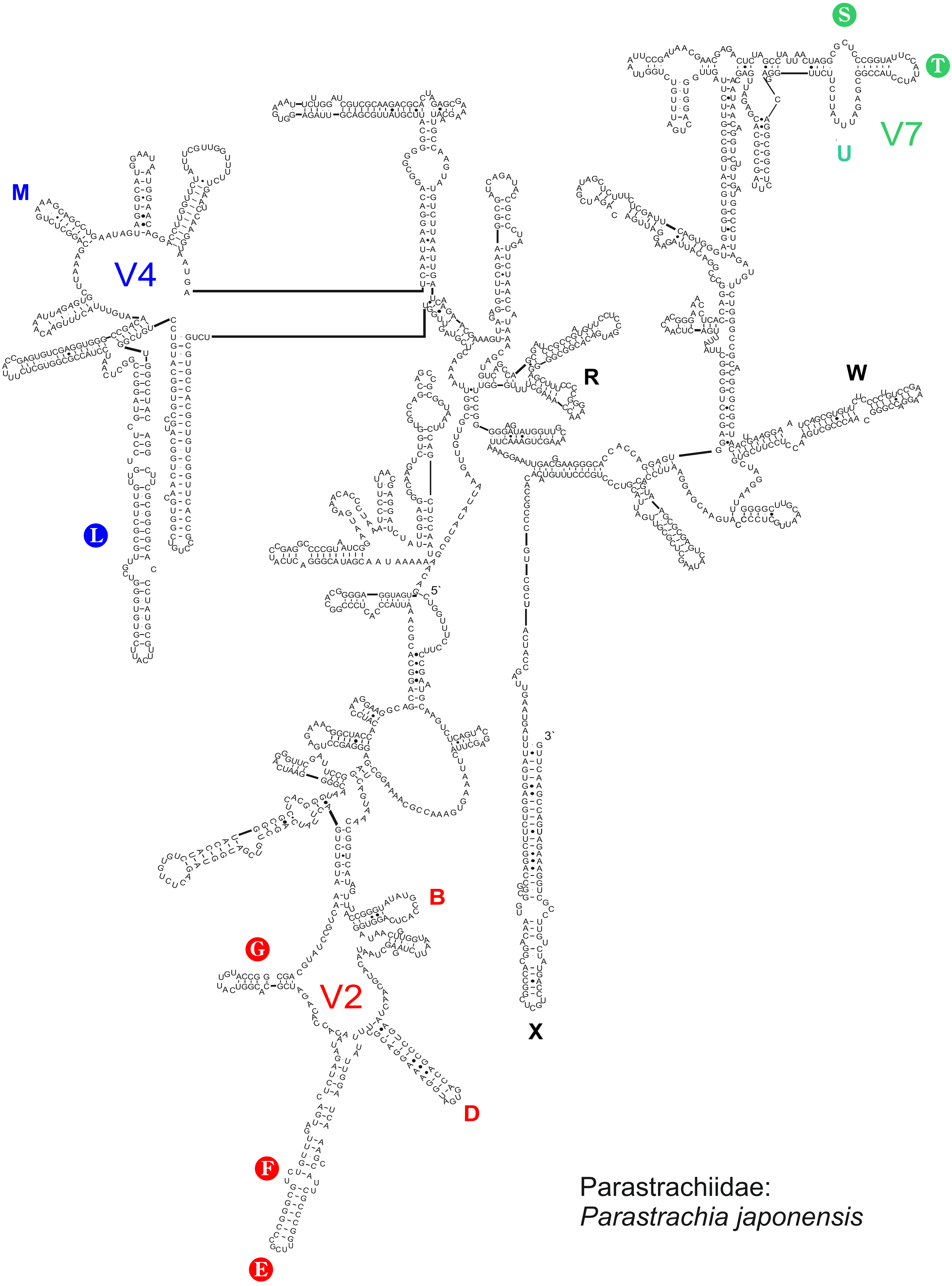

Parastrachiidae:
Parastrachia japonensis

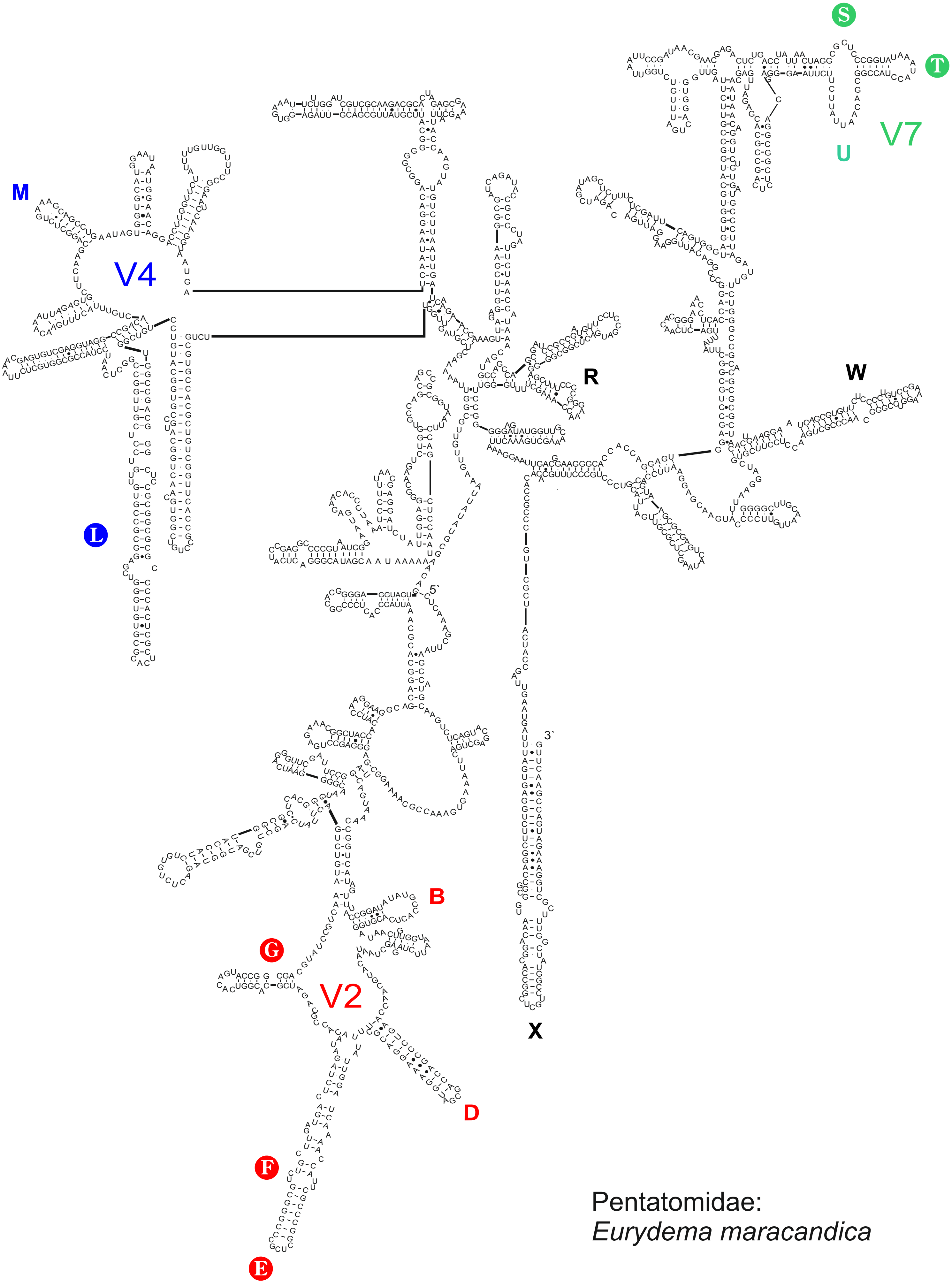

Pentatomidae:
Eurydema maracandica

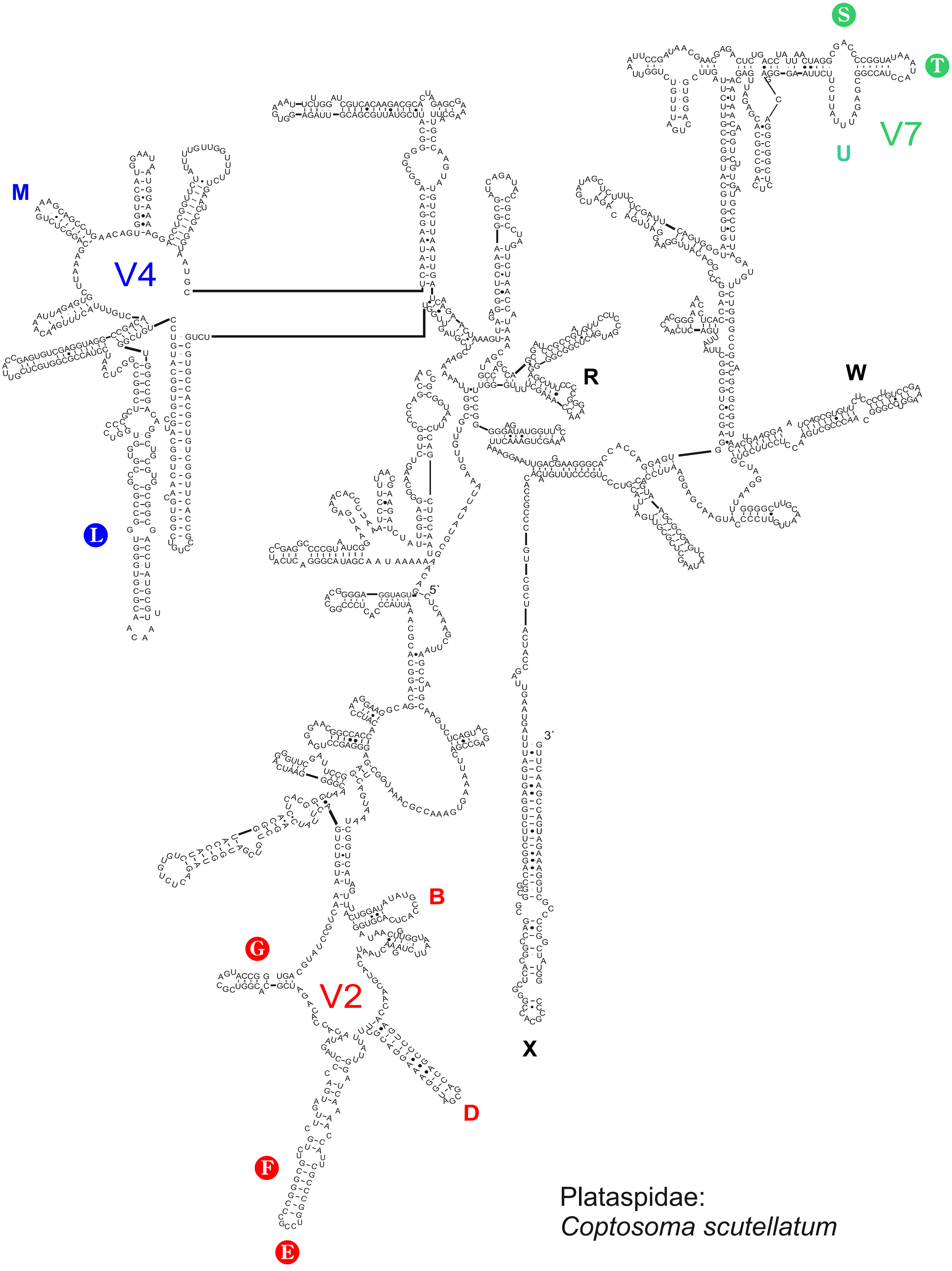

Plataspidae:
Coptosoma scutellatum

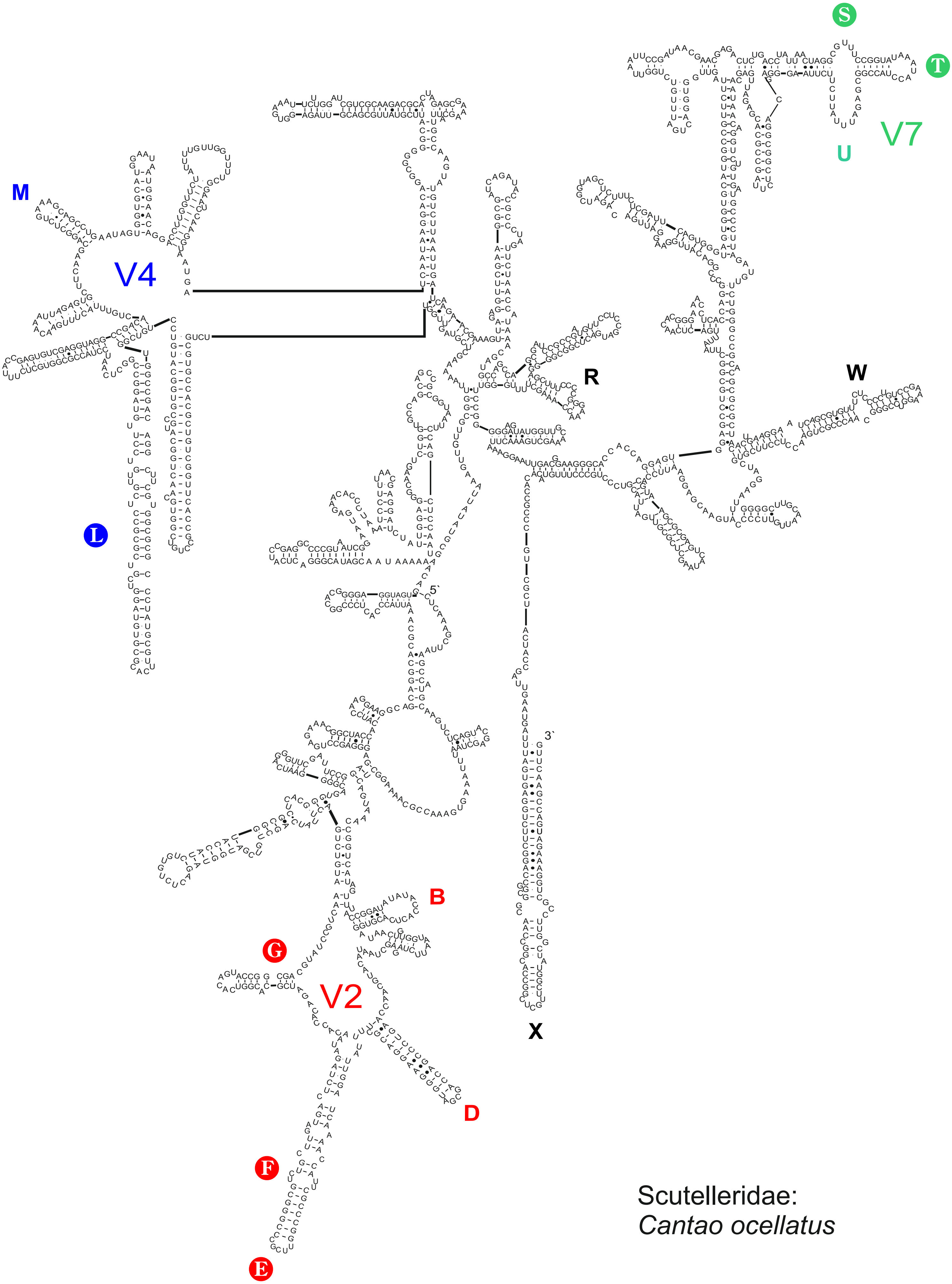

Scutelleridae:
Cantao ocellatus

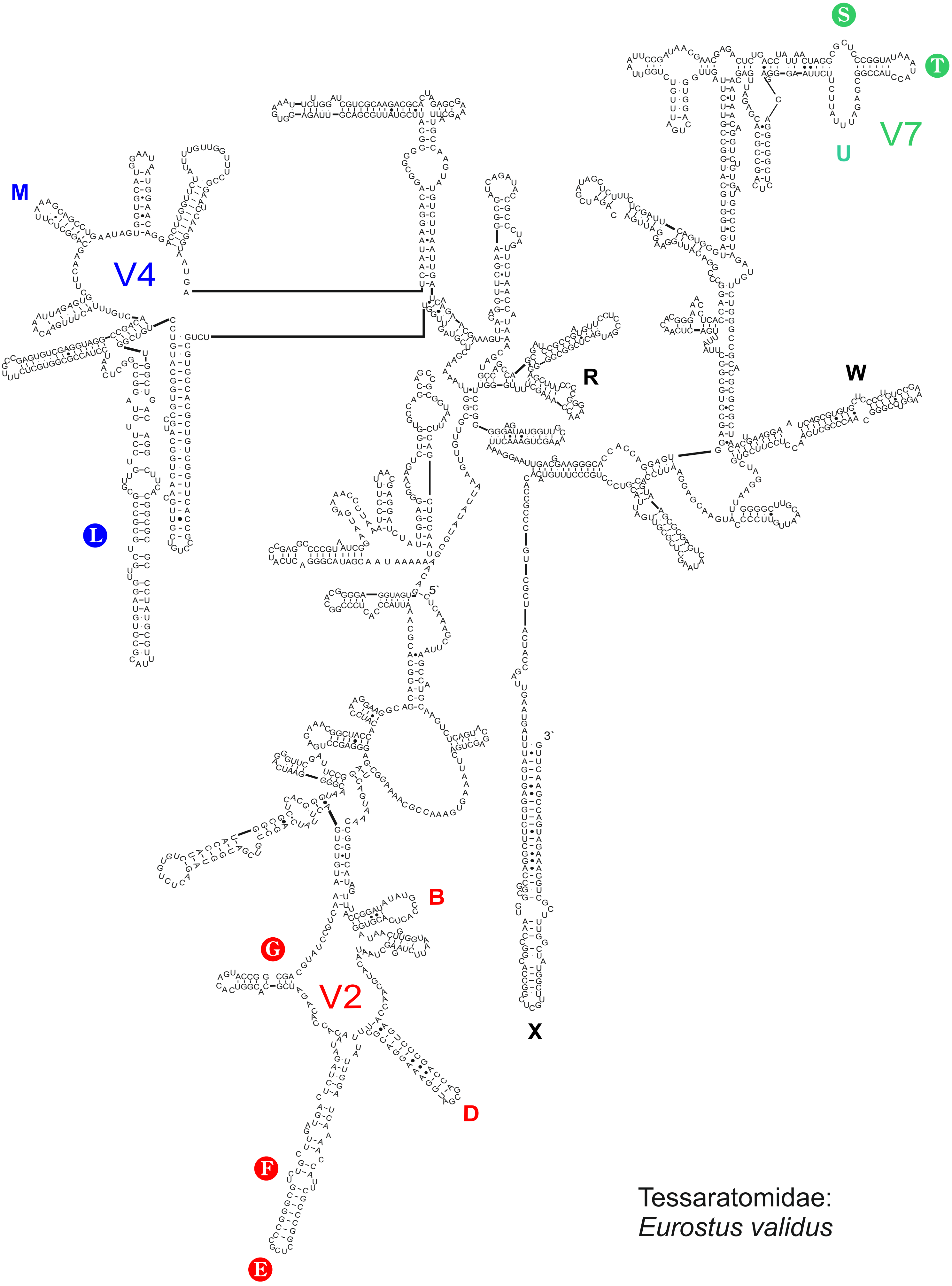

Tessaratomidae:
Eurostus validus

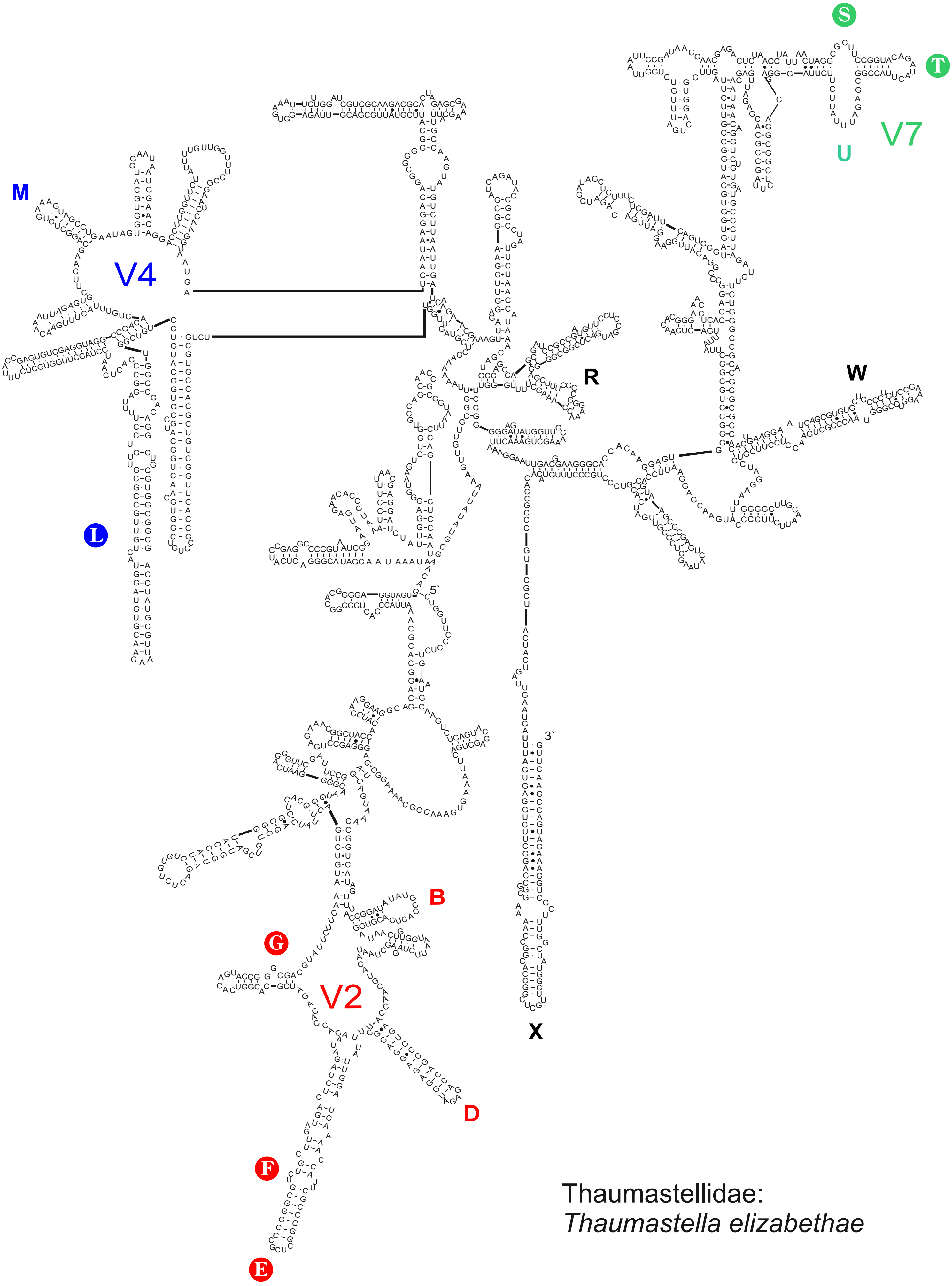

Thaumastellidae:
Thaumastella elizabethae

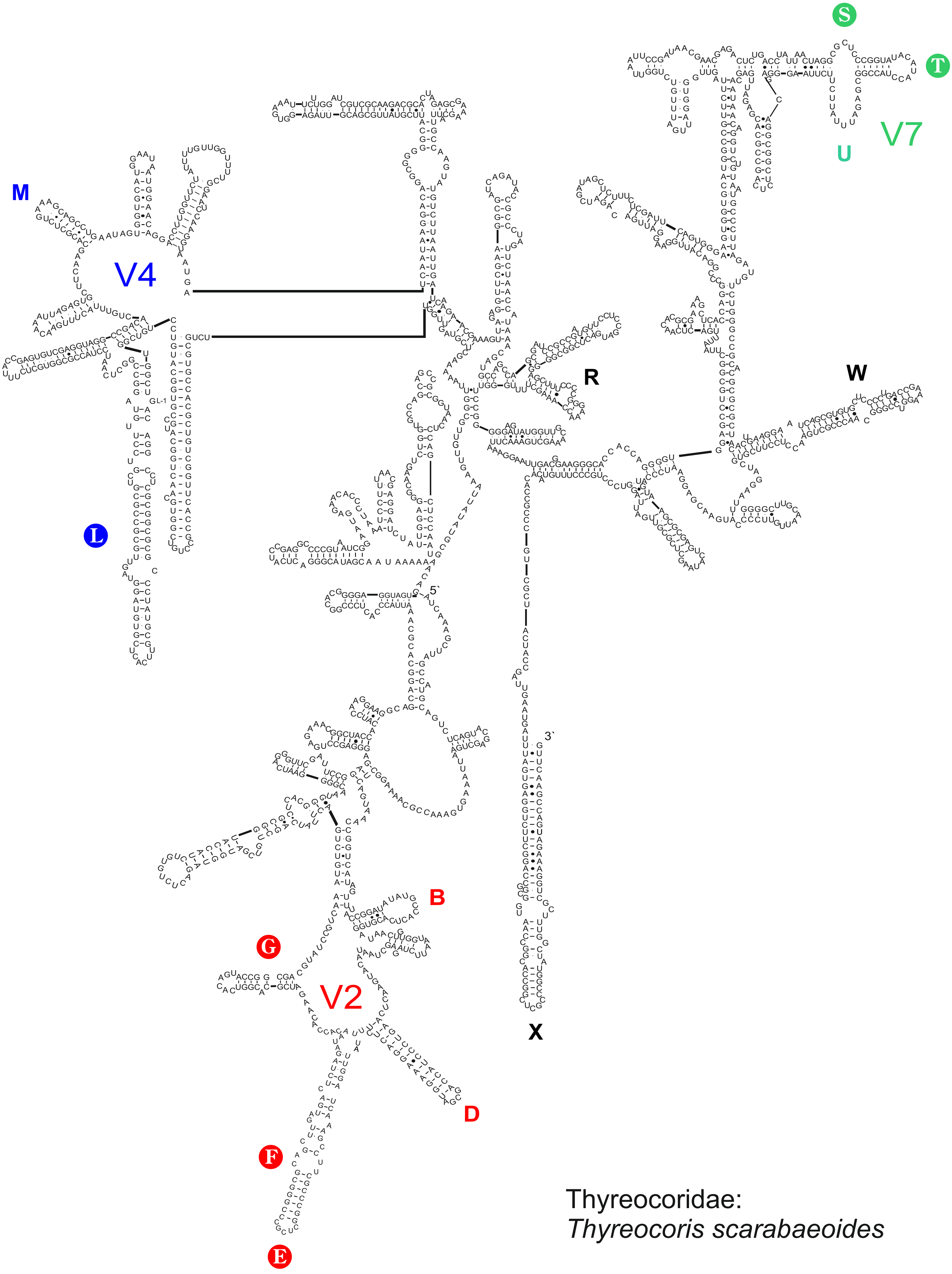

Thyreocoridae:
Thyreocoris scarabaeoides

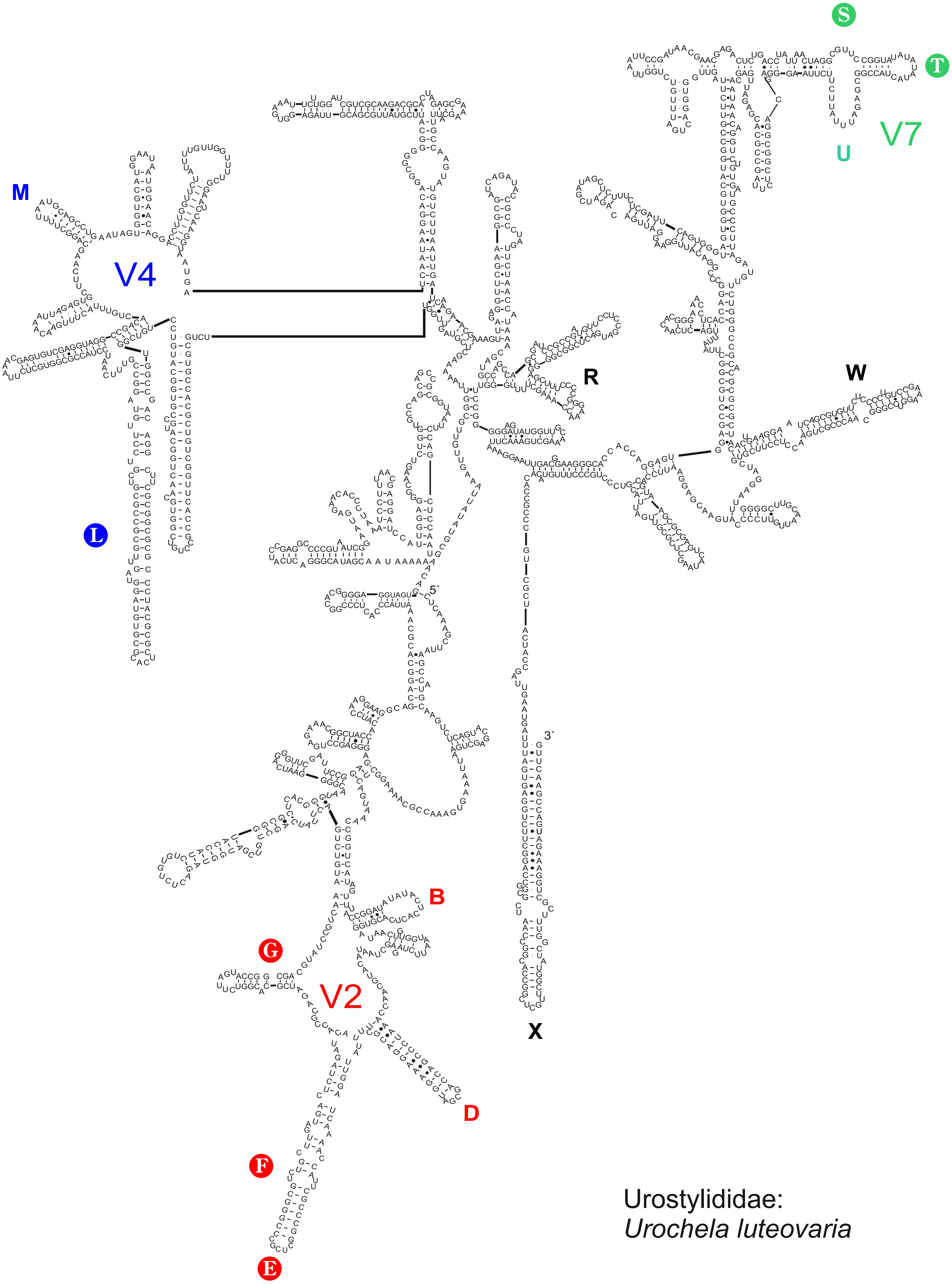

Urostylididae:
Urochela luteovaria
