# Supplementary material for: Molecular Apomorphies in the Secondary and Tertiary Structures of Length-Variable Regions (LVRs) of 18S rRNA Shed Light on the Systematic Position of the Family Thaumastellidae (Hemiptera: Heteroptera: Pentatomoidea)
Source: Int J Mol Sci. 2023 Apr 24;24(9):7758. doi: 10.3390/ijms24097758 (PMC10178826; doi:10.3390/ijms24097758)
Supplement: Supplementary file 1 [file ijms-24-07758-s001.zip › FILE S3.pdf]

FILE S3. Secondary structure models of the LVR L for all consensus species presented in Table 3.

Table 3. The number of nucleotides of the length-variable region L (LVR L) of the analyzed taxa (for details, see the text of the paper).

Taxon group	Consensus species	Total length	Number of nucleotides of the LVR L fragments					
			L2	LA (A1+A2)	LB (B1+B2)	LC (C1+C2)	LD (D1+D2)	LE (E1+E2)
outgroup	*R. pedestris*	75	4	14 (7+7)	16 (9+7)	14 (7+7)	9 (6+3)	18 (9+9)
Acanthosomatidae	*E. interstinctus*	74	4	15 (7+8)	16 (9+7)	14 (7+7)	9 (6+3)	16 (8+8)
Canopidae	*Canopus* sp.	73	4	14 (7+7)	16 (9+7)	14 (7+7)	9 (6+3)	16 (8+8)
Cydnidae: Cydninae	*F. pygmaeus*	74	4	14 (7+7)	16 (9+7)	15 (8+7)	9 (6+3)	16 (8+8)
Cydnidae: Sehirinae	*A. biguttatus*	73	6	12 (6+6)	18 (10+8)	14 (7+7)	9 (6+3)	14 (7+7)
Dinidoridae	*Megymenum* sp.	73	4	14 (7+7)	16 (9+7)	12 (6+6)	11 (7+4)	16 (8+8)
Lestoniidae	*L. haustorifera*	75	4	14 (7+7)	20 (11+9)	13 (6+7)	8 (5+3)	16 (8+8)
Parastrachiidae	*P. japonensis*	73	6	12 (6+6)	18 (10+8)	14 (7+7)	9 (6+3)	14 (7+7)
Pentatomidae	*E. maracandica*	73	4	14 (7+7)	16 (9+7)	12 (6+6)	11 (7+4)	16 (8+8)
Plataspidae	*C. scutellatum*	77	4	18 (9+9)	14 (10+4)	16 (8+8)	10 (5+5)	15 (7+8)
Scutelleridae	*C. ocellatus*	73	4	14 (7+7)	16 (9+7)	14 (7+7)	9 (6+3)	16 (8+8)
Tessaratomidae	*E. validus*	73	4	14 (7+7)	16 (9+7)	12 (6+6)	11 (7+4)	16 (8+8)
Thaumastellidae	*T. elizabethae*	75	3	17 (9+8)	11 (6+5)	16 (8+8)	8 (5+3)	20 (10+10)
Thyreocoridae	*T. scarabaeoides*	74	6	14 (7+7)	17 (10+7)	14 (7+7)	9 (6+3)	14 (7+7)
Urostylididae	*U. luteovaria*	73	4	14 (7+7)	16 (9+7)	14 (7+7)	9 (6+3)	16 (8+8)

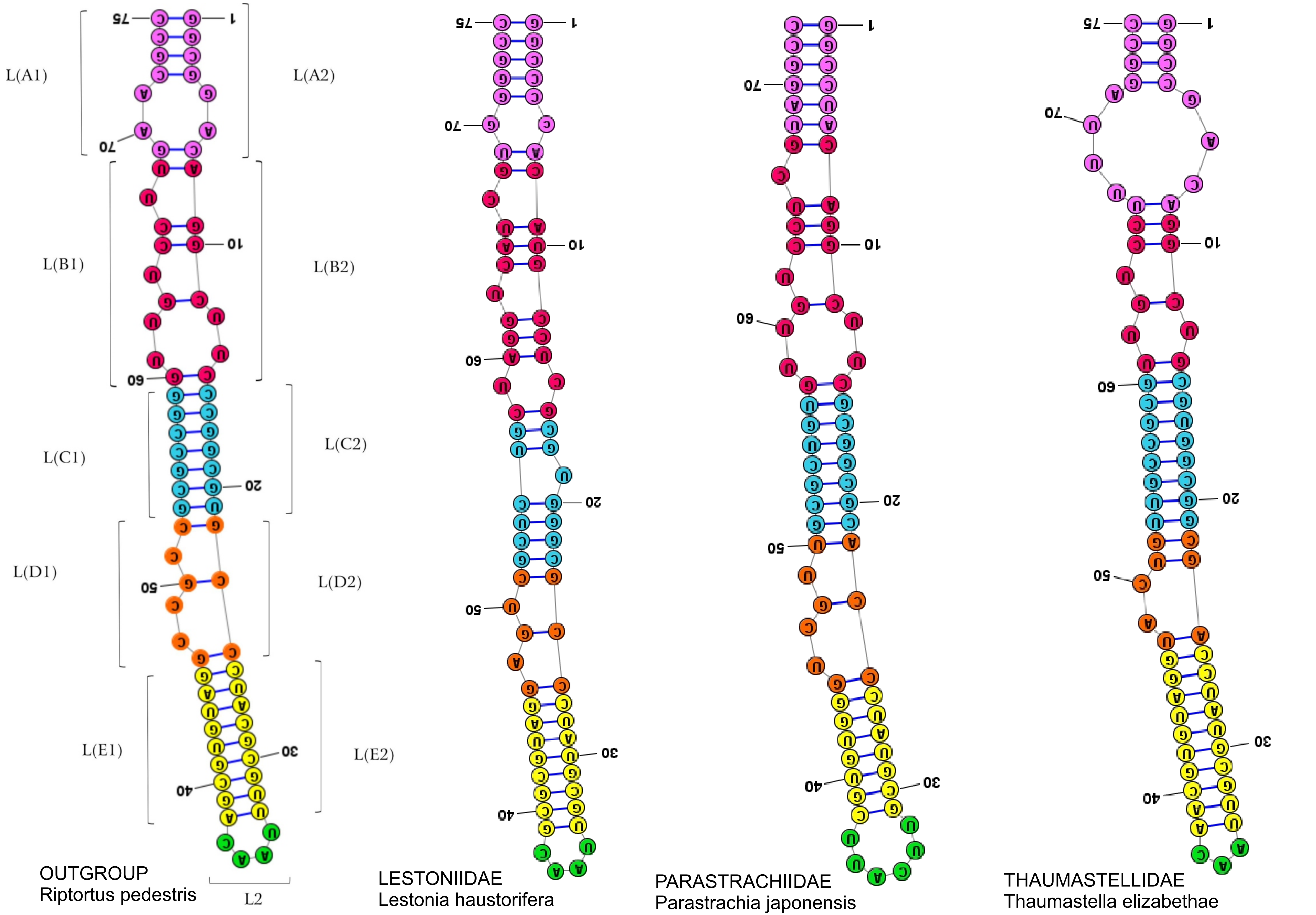
L(A1)
L(A2)
L(B1)
L(B2)
L(C1)
L(C2)
L(D1)
L(D2)
L(E1)
L(E2)
L2
OUTGROUP
Riptortus pedestris
LESTONIIDAE
Lestonia haustorifera
PARASTRACHIIDAE
Parastrachia japonensis
THAUMASTELLIDAE
Thaumastella elizabethae

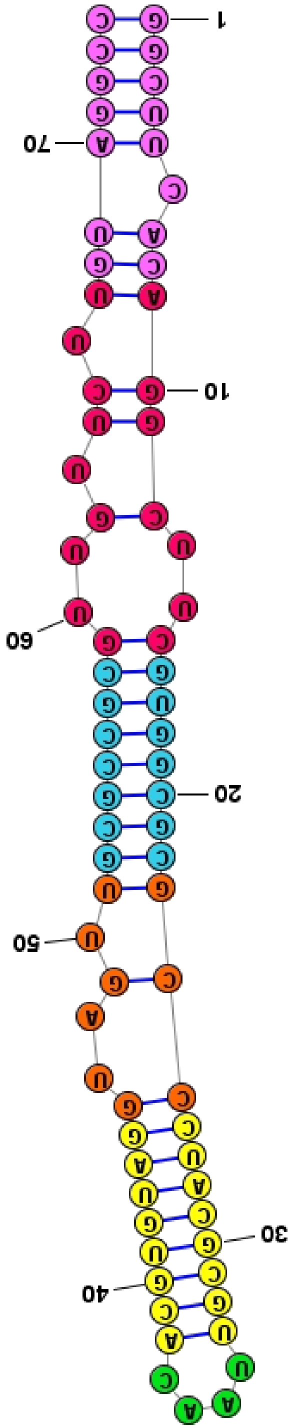

ACANTHOSOMATIDAE
Elasmostethus interstinctus

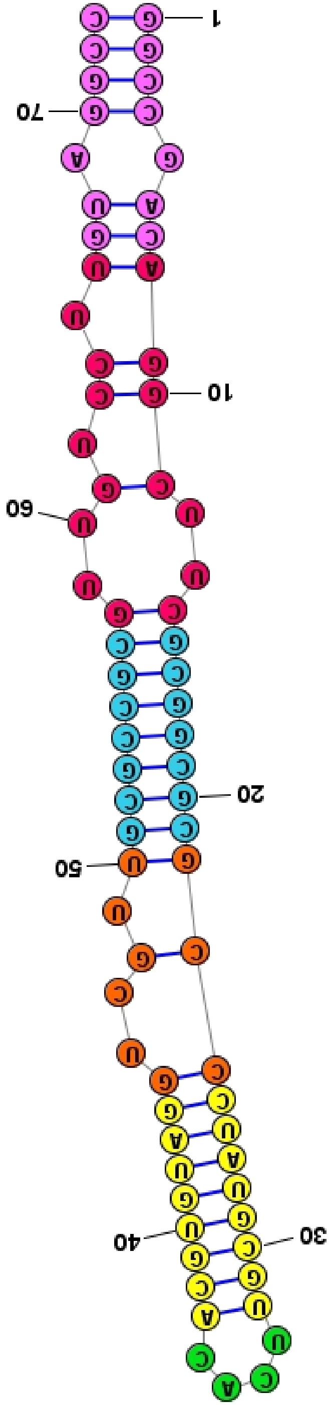

CANOPIDAE
Canopus sp.

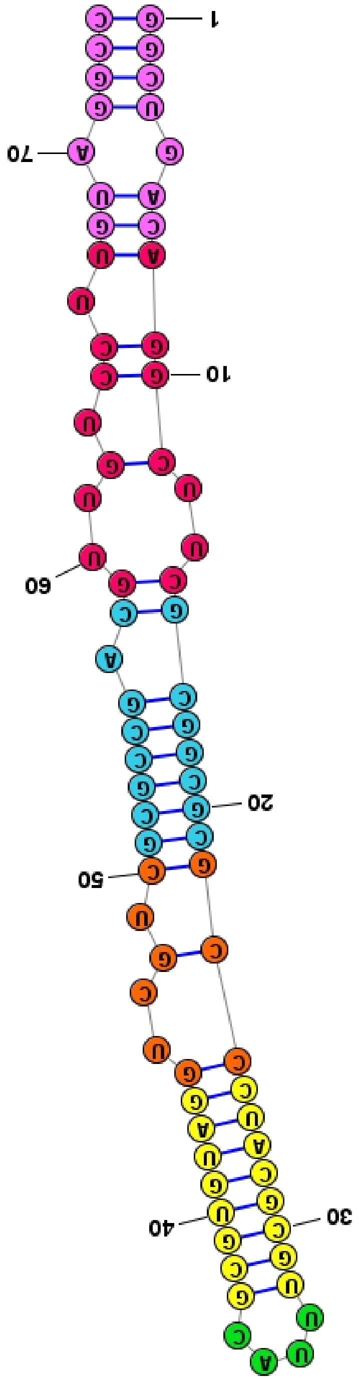

CYDNIDAE: Cydninae
Fromundus pygmaeus

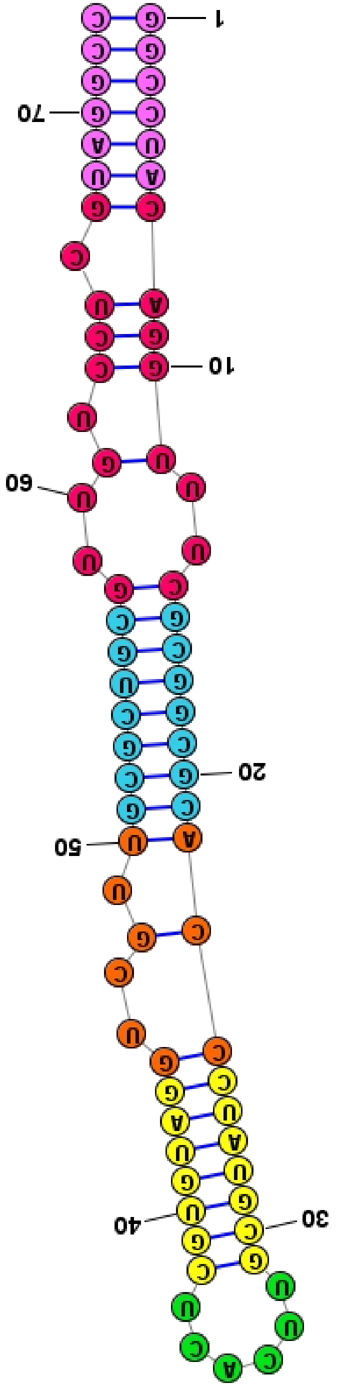

CYDNIDAE: Sehirinae
Adomerus biguttatus

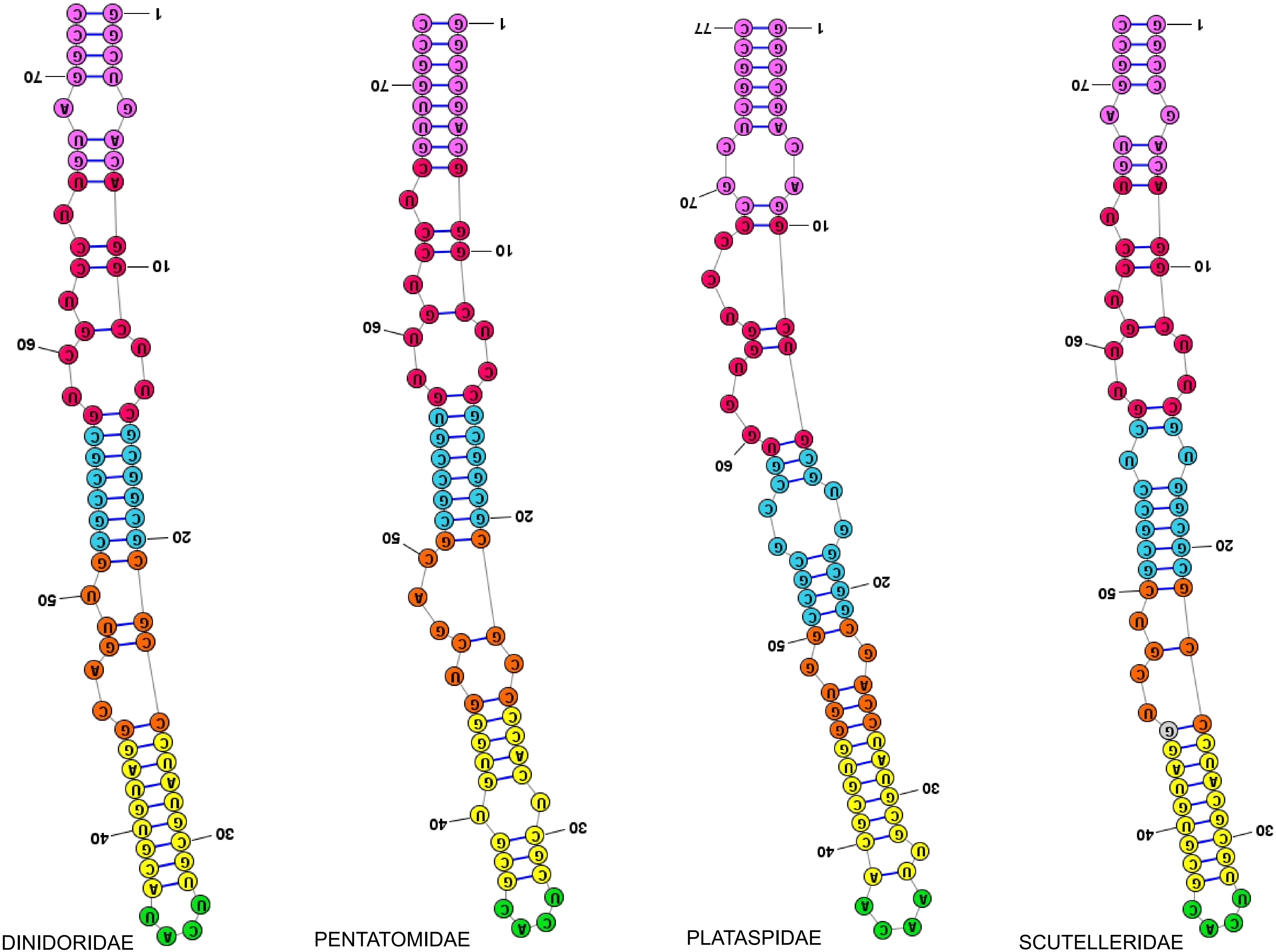

DINIDORIDAE
Megymenum sp.

PENTATOMIDAE
Eurydema maracandica

PLATASPIDAE
Coptosoma scutellatum

SCUTELLERIDAE
Cantao ocellatus

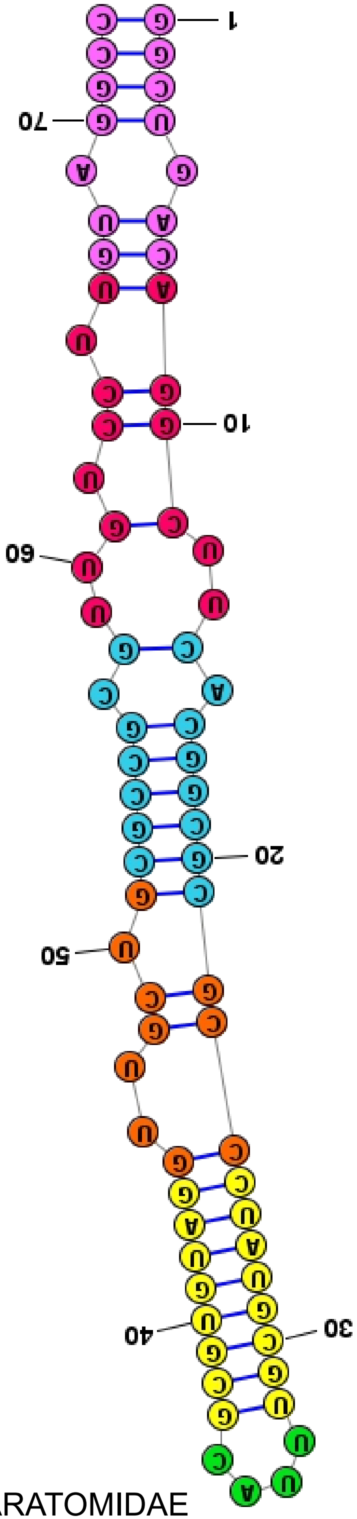

TESSARATOMIDAE
Eurostus validus

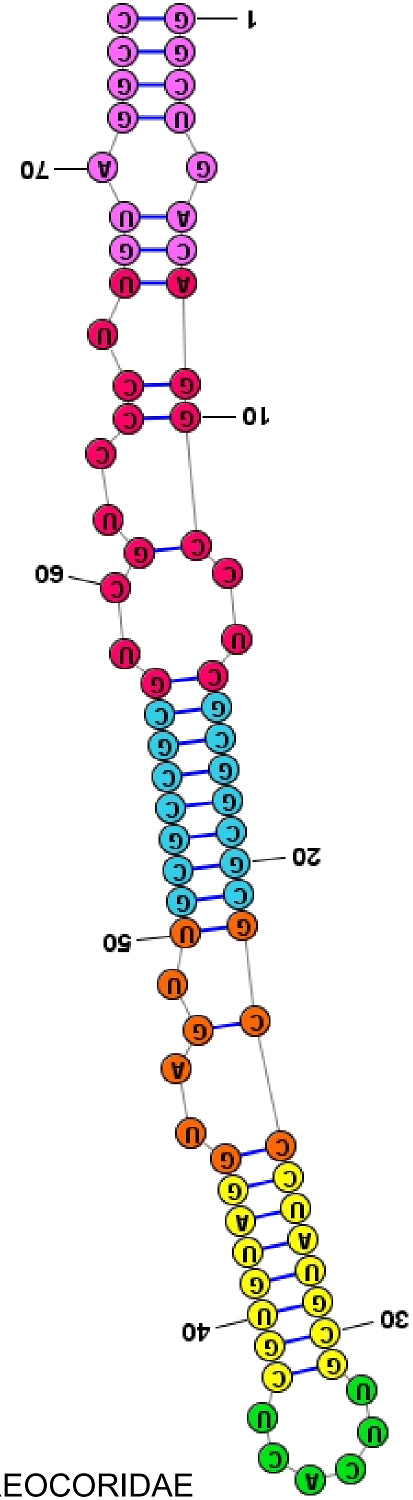

THYREOCORIDAE
Thyreocoris scarabaeoides

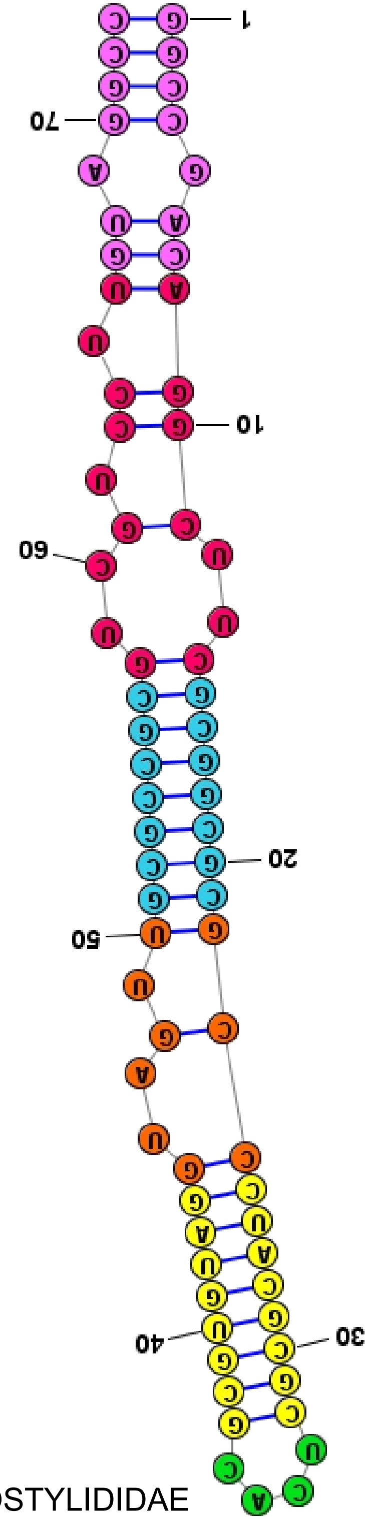

UROSTYLIDIDAE
Urochela luteovaria
