# Supplementary material for: Molecular Apomorphies in the Secondary and Tertiary Structures of Length-Variable Regions (LVRs) of 18S rRNA Shed Light on the Systematic Position of the Family Thaumastellidae (Hemiptera: Heteroptera: Pentatomoidea)
Source: Int J Mol Sci. 2023 Apr 24;24(9):7758. doi: 10.3390/ijms24097758 (PMC10178826; doi:10.3390/ijms24097758)
Supplement: Supplementary file 1 [file ijms-24-07758-s001.zip › FILE S4.pdf]

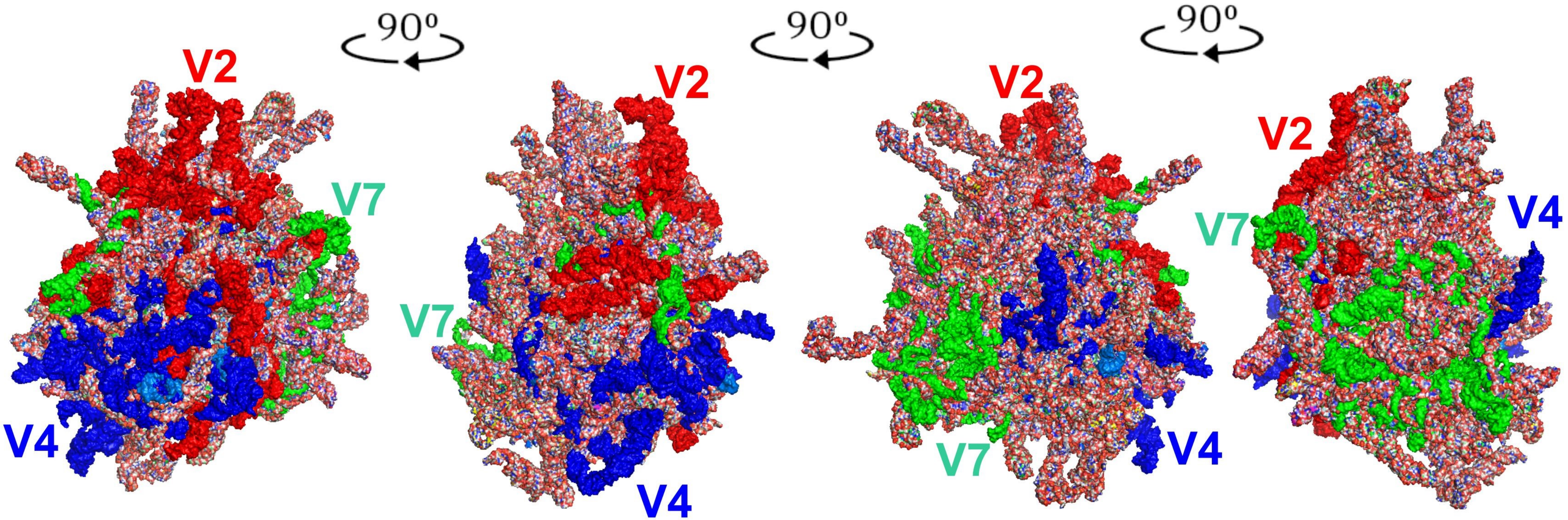

Sequences of all fifteen consensus species aligned to the outgroup (*Riptortus pedestris*) sequence

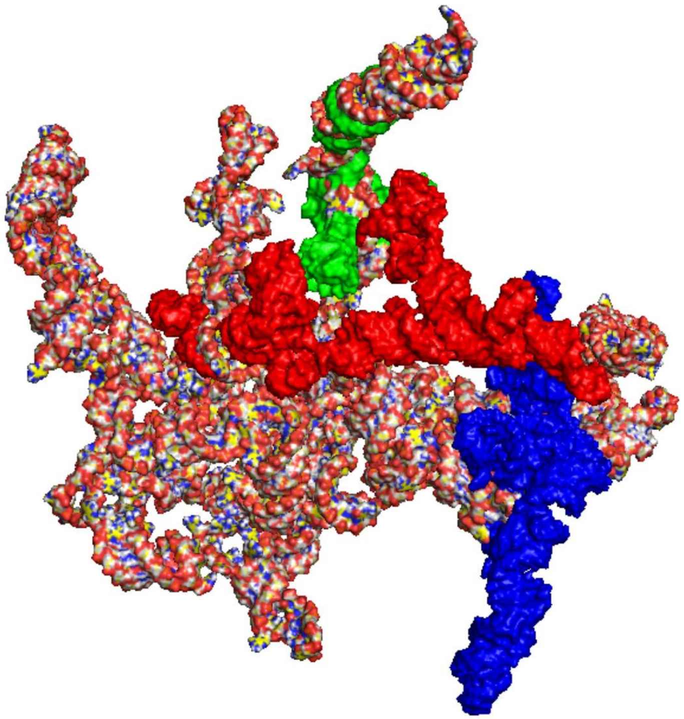

Adomerus biguttatus
(Cydnidae: Sehirinae)

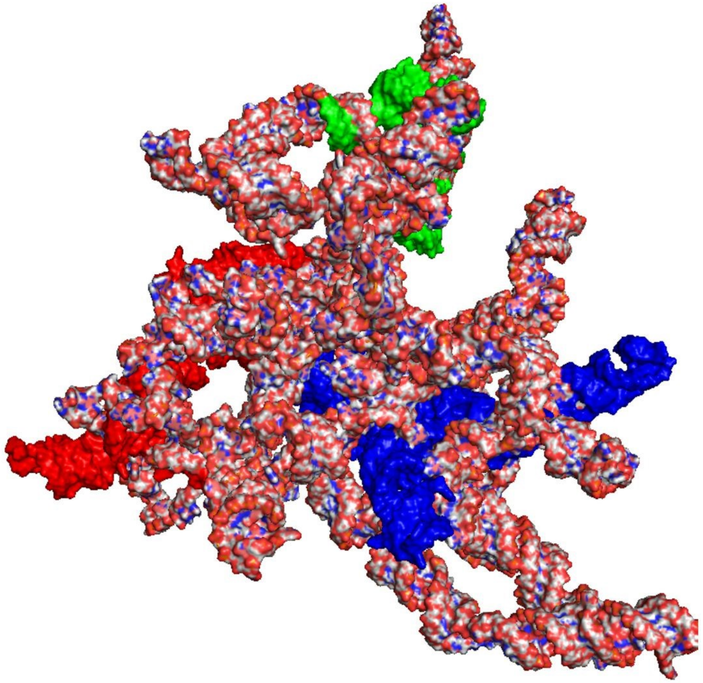

Canopus sp.
(Canopidae)

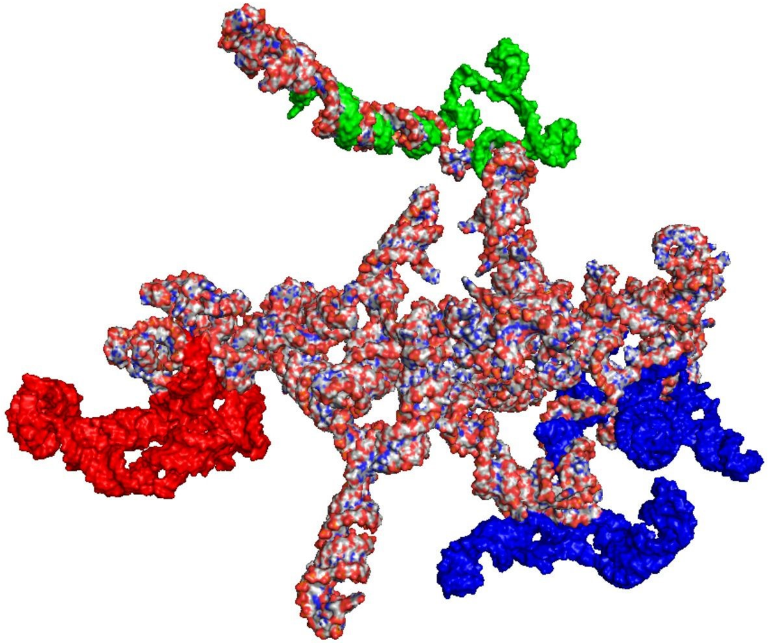

Cantao ocellatus
(Scutelleridae)

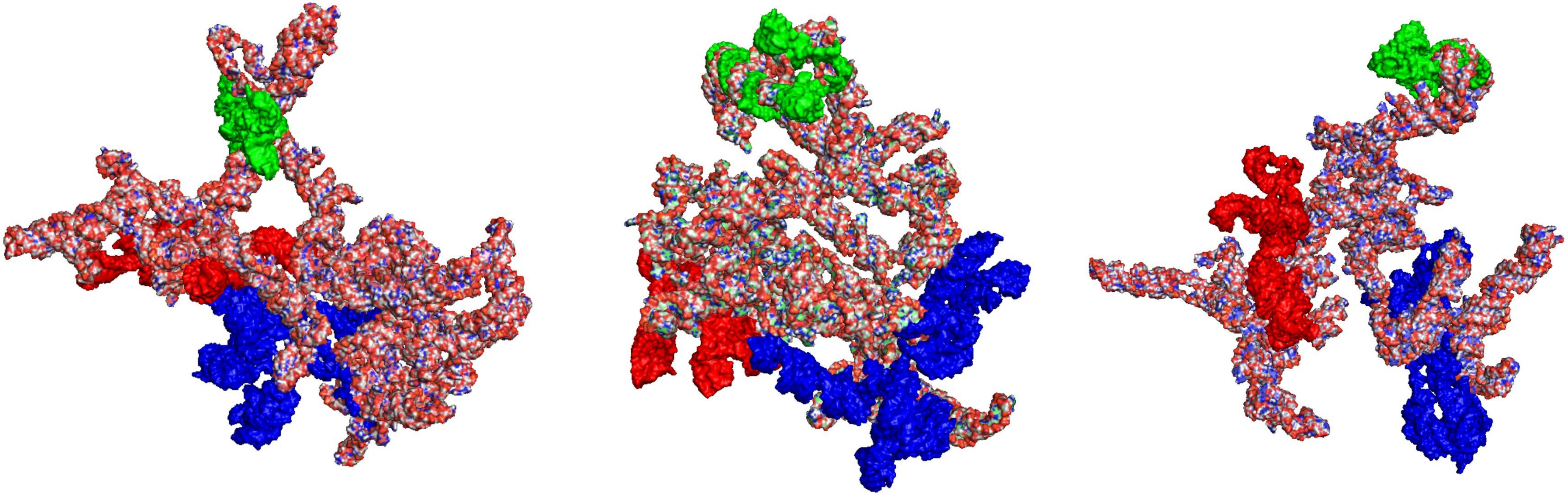

Coptosoma scutellatum (Plataspidae) *Elasmostethus interstinctus* (Acanthosomatidae) *Eurostus validus* (Tessaratomidae)

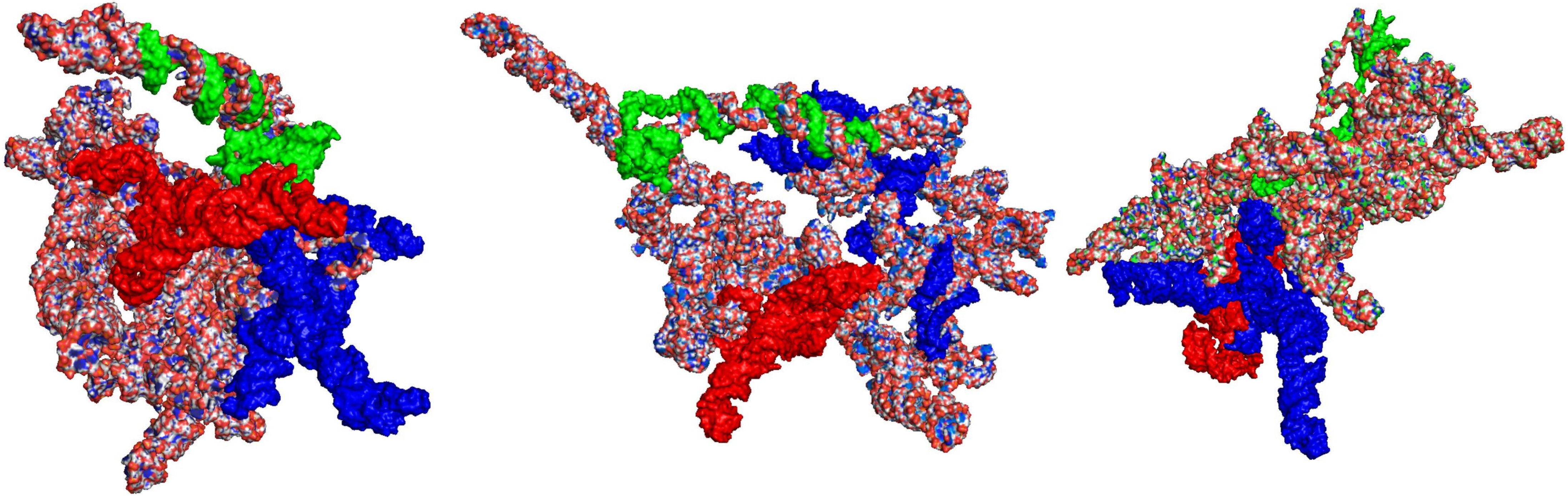

Eurydema maracandica (Pentatomidae)

Fromundus pygmaeus (Cydnidae: Cydninae)

Lestonia haustorifera (Lestoniidae)

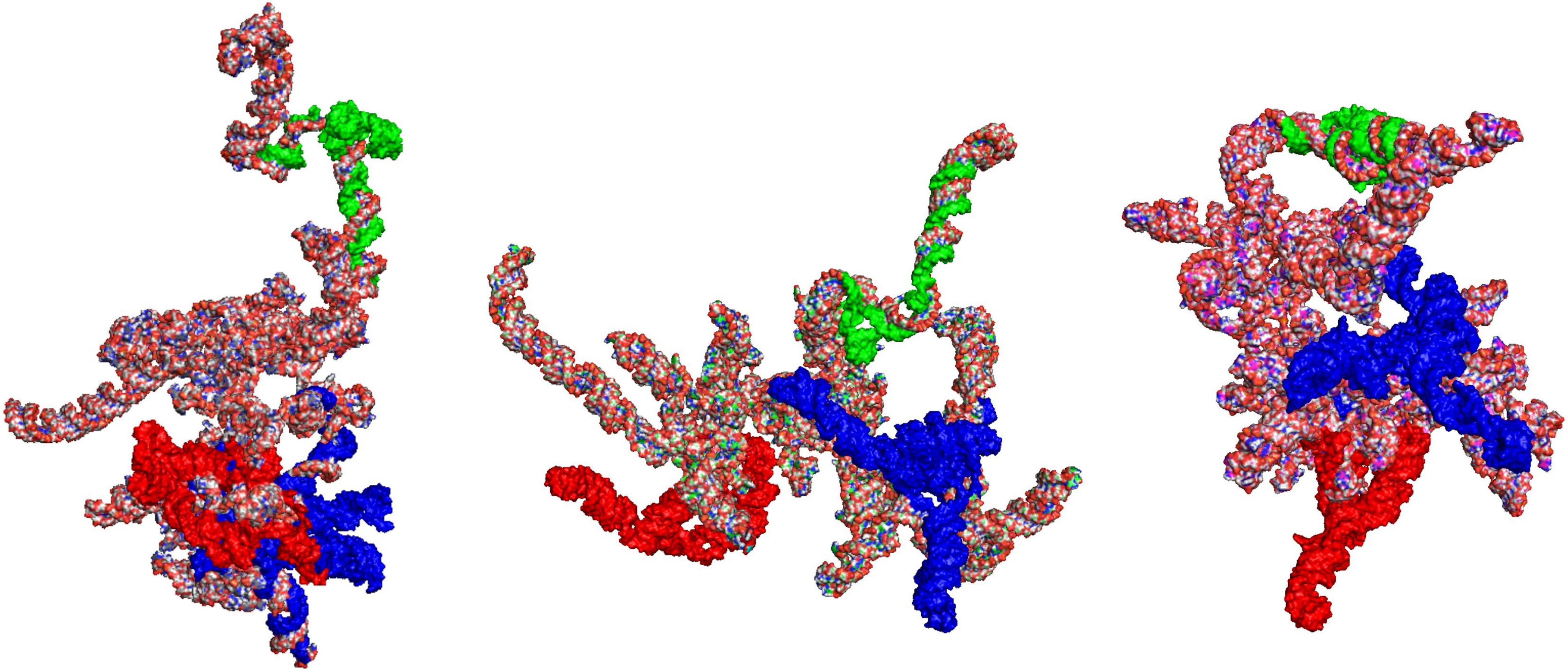

Megymenum sp.
(Dinidoridae)

Riptortus pedestris
(outgroup)

Parastrachia japonensis
(Parastrachiidae)

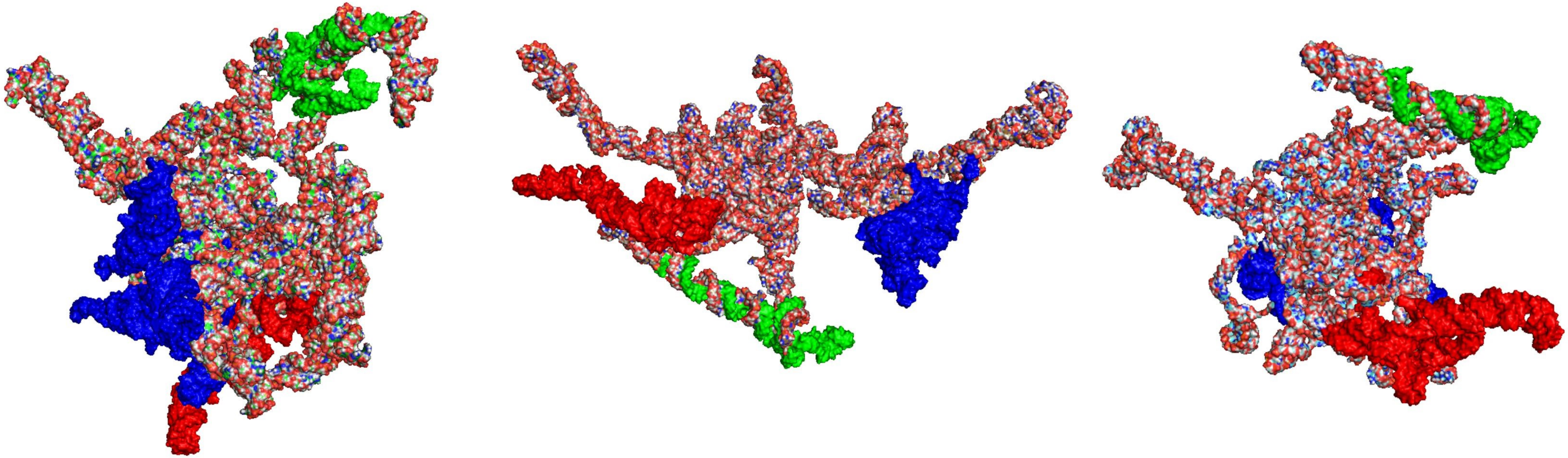

Thaumastella elizabethae (Thaumastellidae) | *Thyreocoris scarabaeoides* (Thyreocoridae) | *Urochela luteovaria* (Urostylididae)
