# Supplementary material for: Molecular Apomorphies in the Secondary and Tertiary Structures of Length-Variable Regions (LVRs) of 18S rRNA Shed Light on the Systematic Position of the Family Thaumastellidae (Hemiptera: Heteroptera: Pentatomoidea)
Source: Int J Mol Sci. 2023 Apr 24;24(9):7758. doi: 10.3390/ijms24097758 (PMC10178826; doi:10.3390/ijms24097758)
Supplement: Supplementary file 1 [file ijms-24-07758-s001.zip › FILE S5.pdf]

FILE S5. Tertiary structure models of the 18S rRNA gene in sole and combined positions. The hypervariable regions are marked in red (V2), blue (V4), and green (V7).

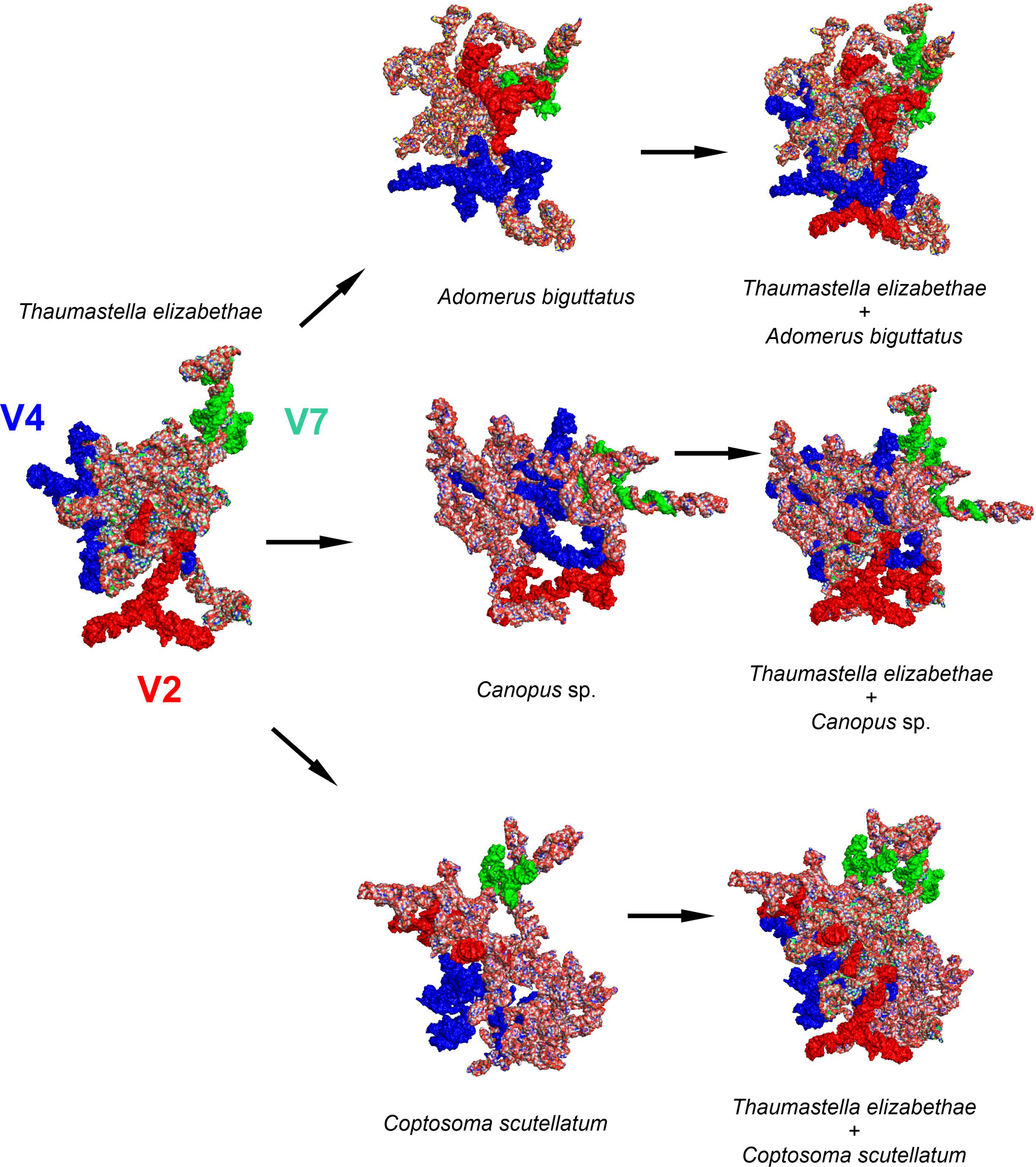

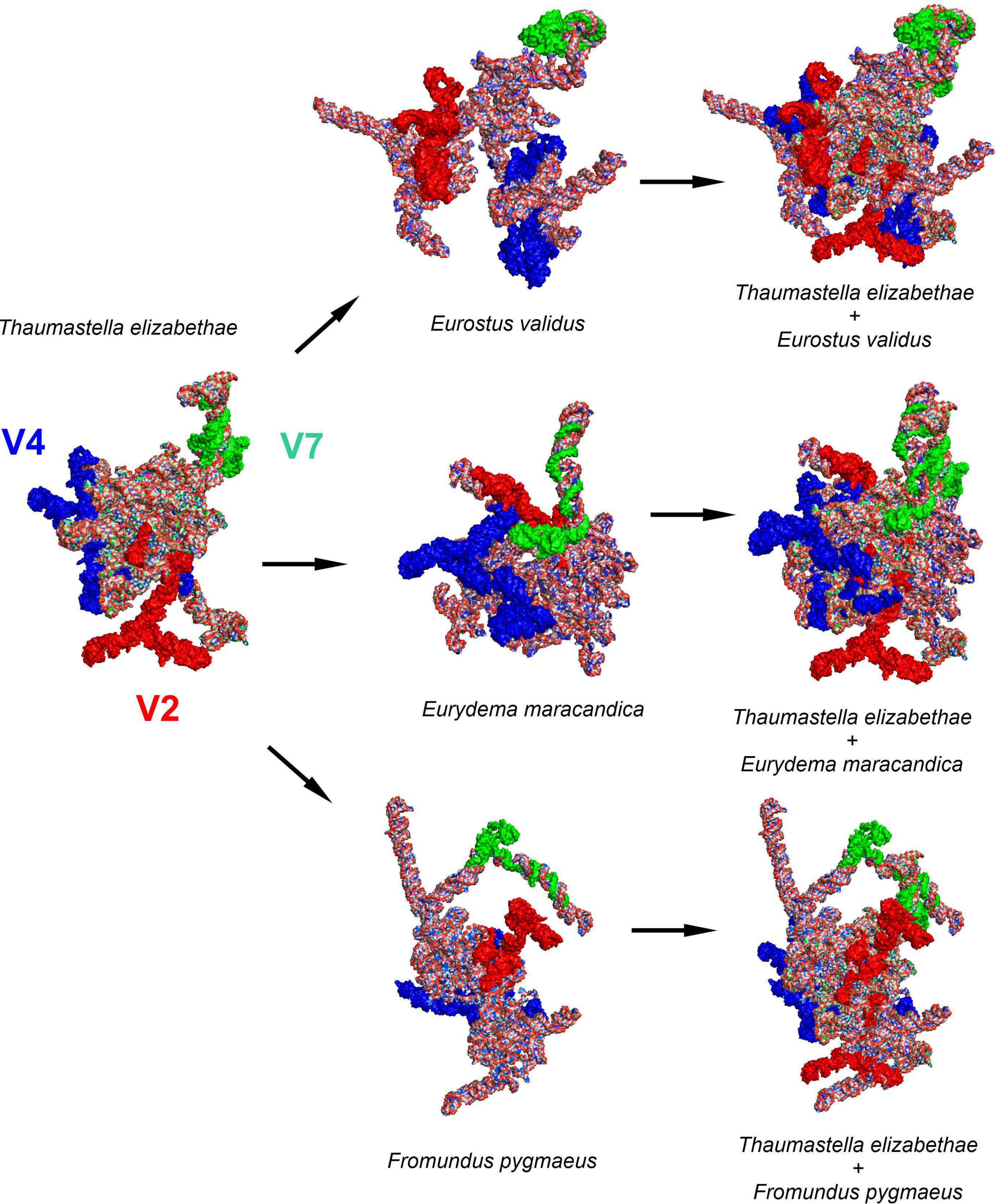
Thaumastella elizabethae
V4
V7
V2
Eurostus validus
Thaumastella elizabethae
+
Eurostus validus
Eurydema maracandica
Thaumastella elizabethae
+
Eurydema maracandica
Fromundus pygmaeus
Thaumastella elizabethae
+
Fromundus pygmaeus

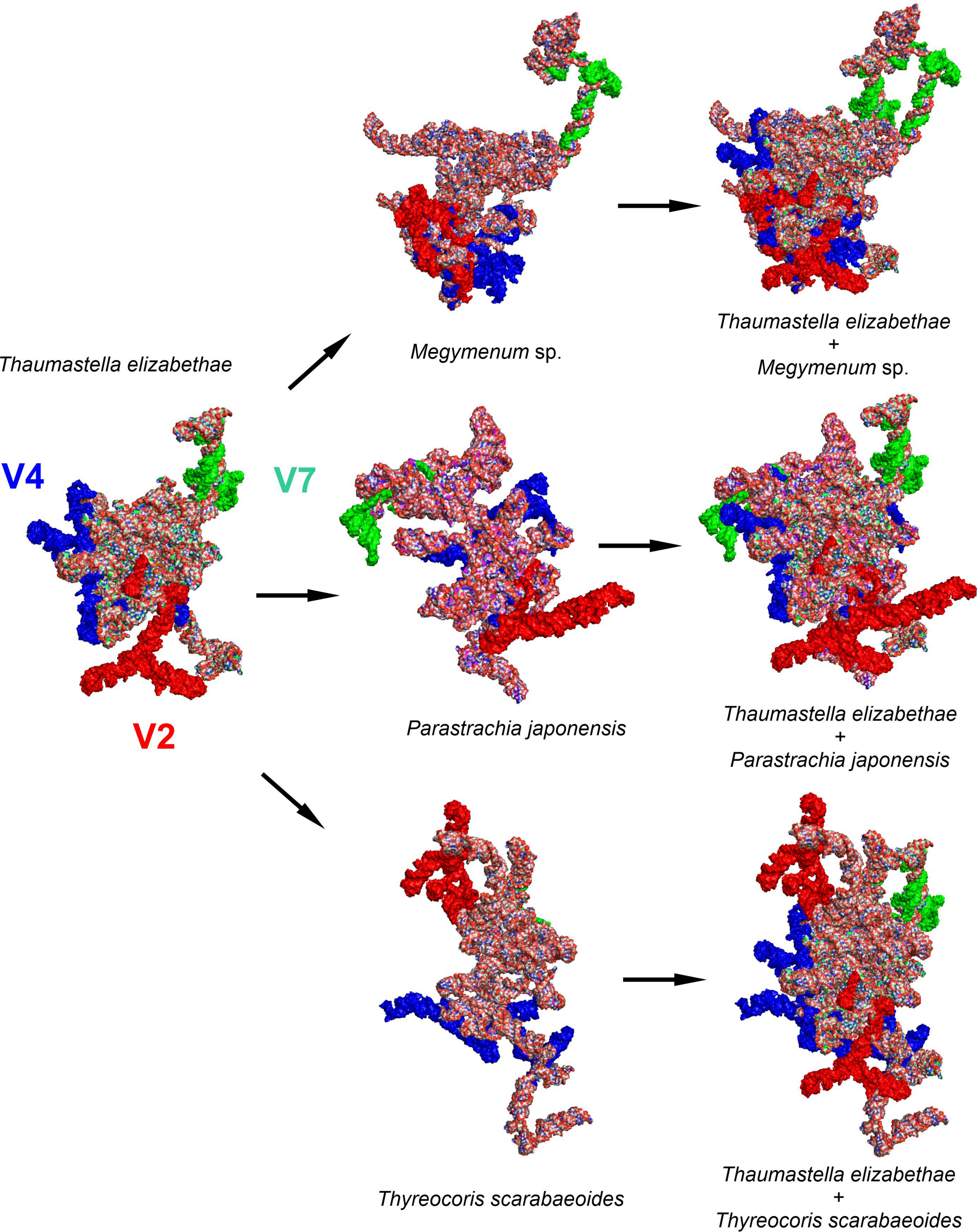
Thaumastella elizabethae
V4
V7
V2
Megymenum sp.
Thaumastella elizabethae
+
Megymenum sp.
Parastrachia japonensis
Thaumastella elizabethae
+
Parastrachia japonensis
Thyreocoris scarabaeoides
Thaumastella elizabethae
+
Thyreocoris scarabaeoides
