# Supplementary material for: Molecular Apomorphies in the Secondary and Tertiary Structures of Length-Variable Regions (LVRs) of 18S rRNA Shed Light on the Systematic Position of the Family Thaumastellidae (Hemiptera: Heteroptera: Pentatomoidea)
Source: Int J Mol Sci. 2023 Apr 24;24(9):7758. doi: 10.3390/ijms24097758 (PMC10178826; doi:10.3390/ijms24097758)

File S6. The LVR L position (marked in light blue) within the hypervariable region V4 in the tertiary structure models of the 18S rRNA gene. The hypervariable regions are marked in red (V2), dark blue (V4), and green (V7). The sequences were not aligned, and each is displayed in such a position that the LVR L can best be seen.

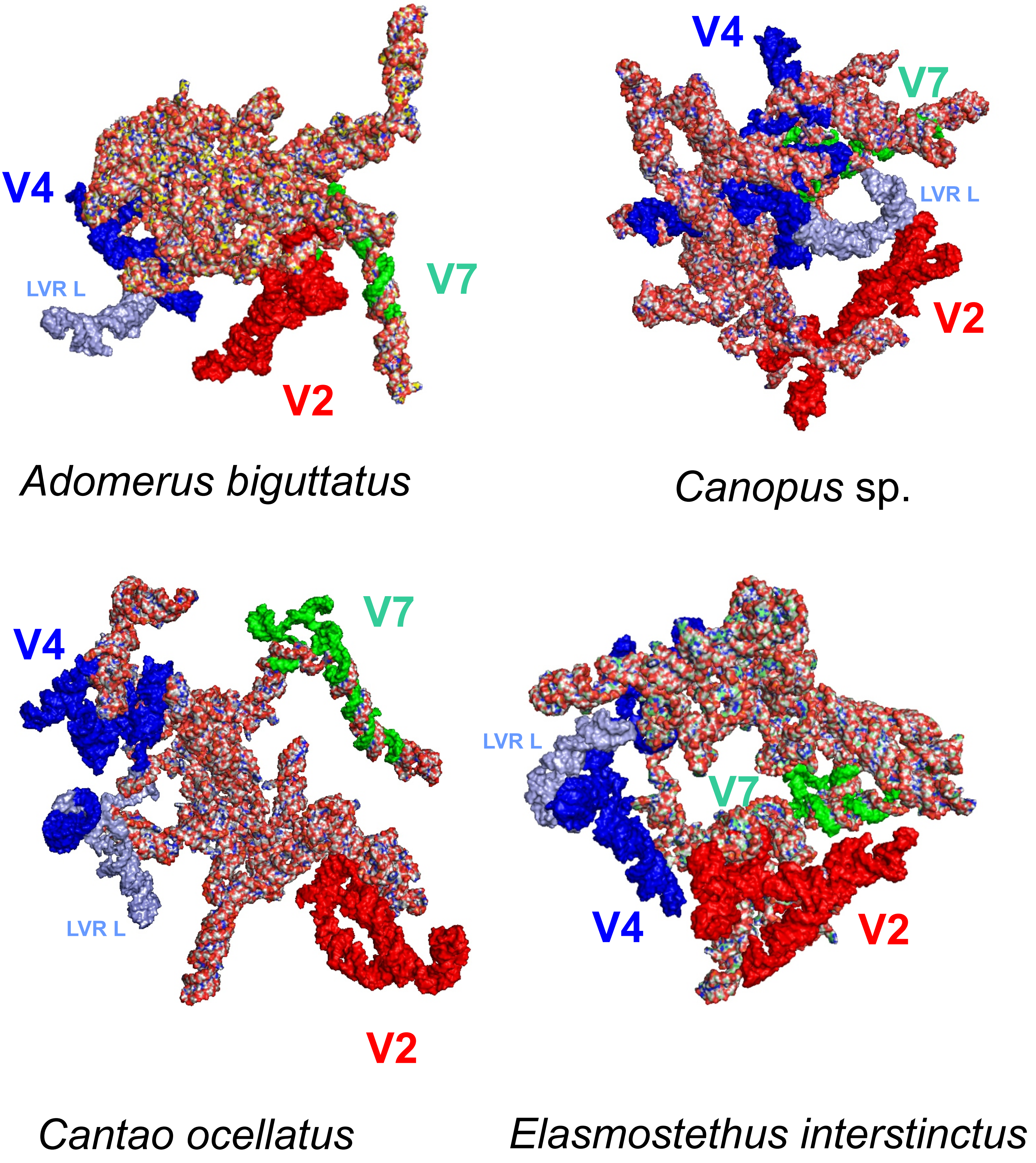

File S6. The LVR L position (marked in light blue) within the hypervariable region V4 in the tertiary structure models of the 18S rRNA gene. The hypervariable regions are marked in red (V2), dark blue (V4), and green (V7). The sequences were not aligned, and each is displayed in such a position that the LVR L can best be seen.

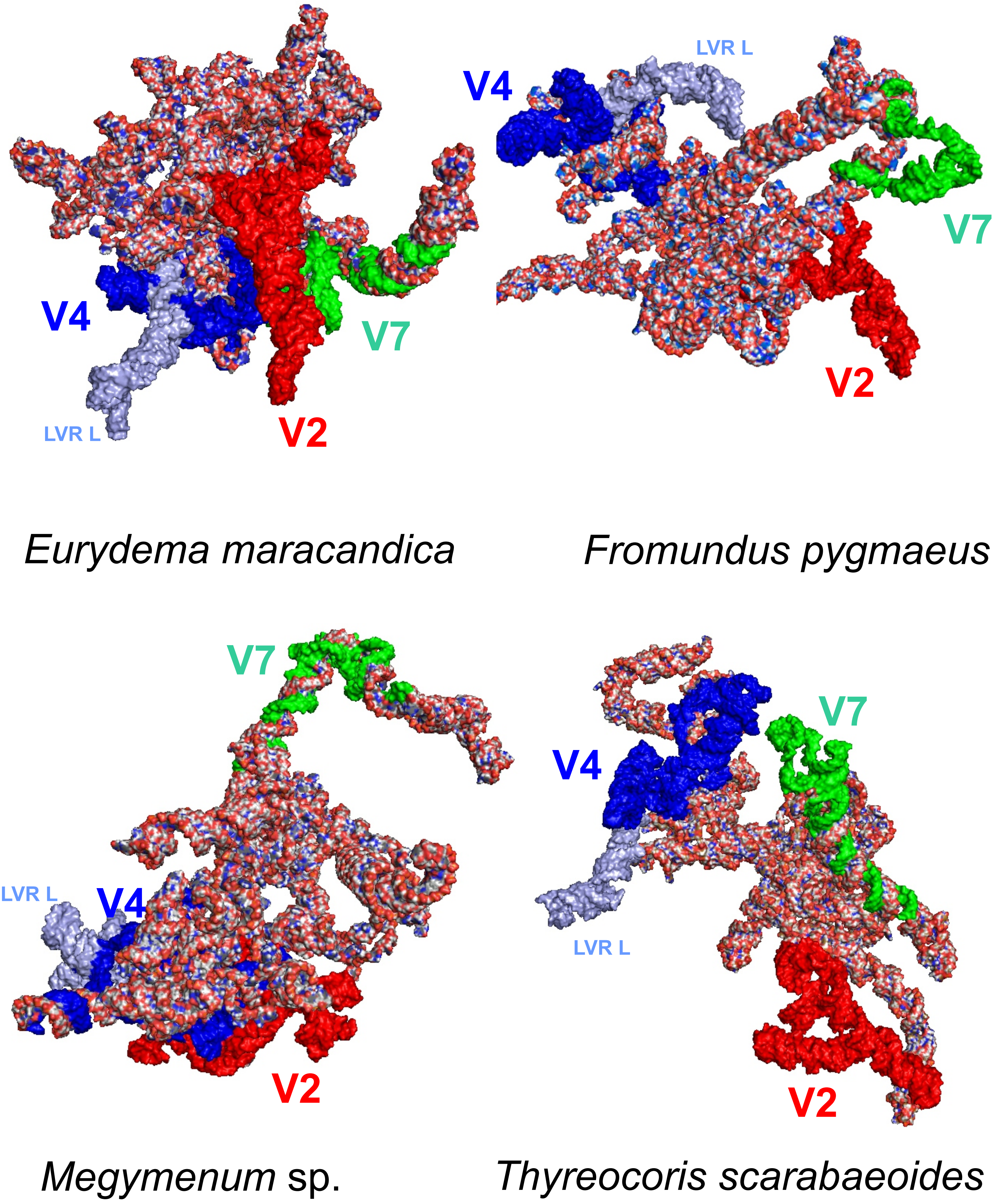

Supplement: Supplementary file 1 [file ijms-24-07758-s001.zip › FILE S6.pdf]
