# Supplementary material for: Molecular Apomorphies in the Secondary and Tertiary Structures of Length-Variable Regions (LVRs) of 18S rRNA Shed Light on the Systematic Position of the Family Thaumastellidae (Hemiptera: Heteroptera: Pentatomoidea)
Source: Int J Mol Sci. 2023 Apr 24;24(9):7758. doi: 10.3390/ijms24097758 (PMC10178826; doi:10.3390/ijms24097758)
Supplement: Supplementary file 1 [file ijms-24-07758-s001.zip › Table S1.pdf]

Table S1. List of species used for the study with GenBank accession numbers for 18S rDNA. The species (marked in read) selected by the computer program RNAstructure ver. 6.3 [1] as exhibiting a secondary structure common to two or more sequences was considered the "consensus species" for these sequences.

Family	Species	Geographic origin	GenBank accesion numbers for 18S rDNA	Source
**Acanthosomatidae**	*Elasmostethus interstinctus* (Linnaeus, 1758)	Poland	KY911197	[2]
	Elasmucha laeviventris Liu, 1919	China	KJ535865	[3]
	Stauralia chloracantha Dallas, 1851	Australia	AY252268	[4]
**Canopidae**	*Canopus* sp.	Peru	AY252229	[4]
	Adrisa magna (Uhler, 1861)	Japan	KY911199	[2]
	Chilocoris confusus Horváth, 1919	Japan	KY911201	[2]
	Cydnus aterrimus (Forster, 1771)	Italy	KY911202	[2]
	Fromundus pygmaeus (Dallas, 1851)	Taiwan	KJ535871	[3]
	Geotomus convexus Hsiao, 1977	Japan	KY911203	[2]
	Macroscytus brunneus (Fabricius, 1803)	Namibia	KY911204	[2]
	Microporus nigrita (Fabricius, 1794)	Poland	KY911205	[2]
	Pangaeus bilineatus (Say, 1825)	Guatemala	KY911207	[2]
	Rhytidoporus indentatus Uhler, 1877	Guam	KY911208	[2]
**Cydnidae: Sehirinae**	*Adomerus biguttatus* (Linnaeus, 1758)	Poland	KY911198	[2]
	Canthophorus niveimarginatus Scott, 1874	Japan	KY911200	[2]
	Ochetostethomorpha secunda J.A. Lis & B. Lis, 2014	Namibia	KY911206	[2]
	Sehirus luctuosus Mulsant et Rey, 1866	Poland	KY911209	[2]
	Tritomegas sexmaculatus (Rambur, 1839)	Poland	KY911210	[2]
**Dinidoridae**	*Cyclopelta obscura* (Lepeletier & Serville, 1828)	China	KJ522641	[3]
	Megymenum sp.	China	KJ535879	[3]
**Lestoniidae**	*Lestonia haustorifera* China, 1955	Australia	KT188471	[3]
**Parastrachiidae**	*Dismegistus sanguineus* (DeGeer, 1778)	South Africa	EF641203	[4]
	Parastrachia japonensis (Scott, 1880)	Japan	EF641204	[4]
**Pentatomidae**	*Eurydema maracandica* Oshanin, 1871	China	KJ535867	[3]
	Graphosoma italicum (O.F. Müller, 1766) [as *G. lineatum* (Linnaeus, 1758) in GenBank	Croatia	KY911211	[2]

	Oechalia schellenbergii (Guérin, 1831)	Australia	EF641205	[4]
	Rhaphigaster nebulosa (Poda, 1761)	Germany	X89495	[5]
**Plataspidae**	*Coptosoma bifarium* Montandon, 1897	China	KJ461259	[6]
	Coptosoma scutellatum (Geoffroy, 1785)	Poland	KY911212	[2]
**Scutelleridae**	*Cantao ocellatus* (Thunberg, 1784)	China	KJ461182	[6]
	Coleotichus costatus (Fabricius, 1787)	Australia	EF641219	[4]
**Tessaratomidae**	*Eurostus validus* Dallas, 1851	China	KJ461181	[6]
**Thaumastellidae**	*Thaumastella elizabethae* Jacobs, 1989	South Africa	EF641221	[4]
	Thaumastella namaquensis Schaefer & Wilcox, 1971	South Africa	EF641222	[4]
**Thyreocoridae**	*Allocoris* sp.	USA	AY252323	[4]
	Galgupha australis McAtee & Malloch, 1933	Bolivia	KY911213	[2]
	Thyreocoris scarabaeoides (Linnaeus, 1758)	Poland	KY911214	[2]
**Urostylididae**	*Tessaromerus licenti* Yang, 1939	China	KJ535883	[3]
	Urochela luteovaria Distant, 1881	China	KJ461205	[6]
	Urostylis chinai Maa, 1947	China	KJ535886	[3]
**Alydidae** (outgroup)	*Leptocorisa acuta* (Thunberg, 1783)	China	AY627322	[7]
	Riptortus pedestris (Fabricius, 1775)*	Japan	AB725684	[9]
**Coreidae** (outgroup)	*Cletus punctiger* (Dallas, 1852)	China	KJ461173	[6]
	Aulacosternum nigrorubrum Dallas, 1852	Australia	AY252258	[8]
**Rhopalidae** (outgroup)	*Stictopleurus punctatonervosus* (Goeze, 1778)	China	KJ461217	[6]
**Stenocephalidae** (outgroup)	*Dicranocephalus alticolus* (Zheng, 1981)	China	KJ461228	[6]

* *Riptortus pedestris* was indicated the "consensus species" for the entire outgroup.

References

1. Reuter, J.S.; Mathews, D.H. RNAstructure: software for RNA secondary structure prediction and analysis. *BMC Bioinformatics* **2010**, *11*, 129.
2. Lis J.A., Ziaja D., Lis B. & Gradowska P.A. 2017. Non-monophyly of the "cydnoid" complex within Pentatomoidea (Hemiptera: Heteroptera) revealed by Bayesian phylogenetic analysis of nuclear rDNA sequences. *Arthropod Systematics & Phylogeny*, **75**, 481–496.

3. Wu Y-Z., Yu S-S., Wang Y-H., Wu H-Y., Li X-R., Men X-Y., Zhang Y-W., Rédei D., Xie Q., Bu W-J. 2016. The evolutionary position of Lestoniidae revealed by molecular autapomorphies in the secondary structure of rRNA besides phylogenetic reconstruction (Insecta: Hemiptera: Heteroptera). *Zoological Journal of the Linnean Society* **1771**: 750–763.
4. Grazia J., Schuh R.T., Wheeler W.C. 2008. Phylogenetic relationships of family groups in Pentatomoidea based on morphology and DNA sequences (Insecta: Heteroptera). *Cladistics* **24**: 1–45.
5. Reumont von B.M., Meusemann K., Szucsich N.U., Dell'Ampio E., Gowri-Shankar V., Bartel D., Simon S., Letsch H.O., Stocsits R.R., Luan Y.X., Wägele J.W., Pass G., Hadrys H., Misof B. 2009. Can comprehensive background knowledge be incorporated into substitution models to improve phylogenetic analyses? A case study on major arthropod relationships. *BMC Evolutionary Biology* **9**: 119.
6. Wang Y-H., Cui Y., Rédei D., Baňař P, Xie Q., Štys P., Damgaard J., Chen P-P., Yi W-B., Wang Y., Dang K., Li Ch-R., Bu W-J. 2016. Phylogenetic divergences of the true bugs (Insecta: Hemiptera: Heteroptera), with emphasis on the aquatic lineages: the last piece of the aquatic insect jigsaw originated in the Late Permian/Early Triassic. *Cladistics* **32**: 390–405.
7. Li H-m., Deng R-q., Wang J-w., Chen Z-y., Jia F-l., Wang X-z. 2005. A preliminary phylogeny of the Pentatomomorpha (Hemiptera: Heteroptera) based on nuclear 18S rDNA and mitochondrial DNA sequences. *Molecular Phylogenetic & Evolution* **37**: 313–326.
8. Wheeler .C., Schuh R.T. 2003. Direct submission to GenBank.
9. Futahashi R., Tanaka K., Tanahashi M., Nikoh N., Kikuchi Y., Lee B.L., Fukatsu T. 2013. Gene expression in gut symbiotic organ of stinkbug affected by extracellular bacterial symbiont. *PLoS ONE* **8 (5)**: e64557.
